# Comparison οf Immune Responses Through Multiparametric T-Cell Cytokine Expression Profile Between Children with Convalescent COVID-19 or Multisystem Inflammatory Syndrome

**DOI:** 10.3390/children11111278

**Published:** 2024-10-23

**Authors:** Filippos Filippatos, Marianna Tzanoudaki, Elizabeth-Barbara Tatsi, Nick Dessypris, Dimitra-Maria Koukou, Chrysa Georgokosta, Vasiliki Syriopoulou, Athanasios Michos

**Affiliations:** 1 Infectious Diseases and Chemotherapy Research Laboratory, First Department of Pediatrics, Medical School, National and Kapodistrian University of Athens, “Aghia Sophia” Children’s Hospital, 11527 Athens, Greece; filippat@med.uoa.gr (F.F.); dkoukou@med.uoa.gr (D.-M.K.); c.georgokosta@paidon-agiasofia.gr (C.G.); vsyriop@med.uoa.gr (V.S.); 2 Department of Immunology and Histocompatibility, “Aghia Sophia” Children’s Hospital, 11527 Athens, Greece; m.tzanoudaki@paidon-agiasofia.gr; 3 University Research Institute for Maternal and Child Health and Precision Medicine, 11527 Athens, Greece; etatsi@med.uoa.gr; 4 Department of Hygiene, Epidemiology and Medical Statistics, Medical School, National and Kapodistrian University of Athens, 11572 Athens, Greece; ndessyp@med.uoa.gr

**Keywords:** SARS-CoV-2, Multisystem Inflammatory Syndrome in Children, biomarker, IL-17, IFNγ

## Abstract

Background/objectives: The immunological pathways that cause Multisystem Inflammatory Syndrome after SARS-CoV-2 infection in children (MIS-C) remain under investigation. Methods: The aim of this study was to prospectively compare the T-cell cytokine expression profile in unvaccinated children with acute MIS-C (MISC_A) before immunosuppression, convalescent MIS-C (one month after syndrome onset, MISC_C), convalescent COVID-19 (one month after hospitalization), and in healthy, unvaccinated controls. The intracellular expression of IL-4, IL-2, IL-17, IFNγ, TNF-α and Granzyme B, and the post SARS-CoV-2-Spike antigenic mix stimulation of T-cell subsets was analyzed by 13-color flow cytometry. Results: Twenty children with a median age (IQR) of 11.5 (7.25–14) years were included in the study. From the comparison of the flow cytometry analysis of the 14 markers of MISC_A with the other three groups (MISC_C, post-COVID-19 and controls), significant differences were identified as follows: 1. CD4^+^IL-17^+^/million CD3^+^: 293.0(256.4–870.9) vs. 50.7(8.4–140.5); *p*-value: 0.03, vs. 96.7(89.2–135.4); *p*-value: 0.03 and vs. 8.7(0.0–82.4); *p*-value: 0.03, respectively; 2. CD8^+^IL-17^+^/million CD3^+^: 335.2(225.8–429.9) vs. 78.0(31.9–128.9) vs. 84.1(0.0–204.6) vs. 33.2(0.0–114.6); *p*-value: 0.05, respectively; 3. CD8^+^IFNγ^+^/million CD3^+^: 162.2(91.6–273.4) vs. 41.5(0.0–77.4); *p*-value: 0.03 vs. 30.3(0.0–92.8); *p*-value: 0.08, respectively. Conclusions: In children presenting with MIS-C one month after COVID-19 infection, T cells were found to be polarized towards IL-17 and IFNγ production compared to those with uncomplicated convalescent COVID-19, a finding that could provide possible immunological biomarkers for MIS-C detection.

## 1. Introduction

SARS-CoV-2 infection causes a wide range of clinical manifestations in children [1]. Despite COVID-19 typically presenting as a mild disease in the pediatric population, certain children may display severe clinical manifestations, requiring hospitalization or developing the most severe condition: Multisystem Inflammatory Syndrome in Children (MIS-C) associated with SARS-CoV-2 infection [1]. Fever, elevated inflammatory markers, the involvement of at least two organ systems, confirmation of SARS-CoV-2 infection or exposure, as well as the exclusion of additional potential causes constitute the criteria for the Centers for Disease Control and Prevention (CDC) and World Health Organization (WHO) case definitions of MIS-C [2,3]. However, the exact activated molecular mechanisms that lead certain pediatric populations to present with MIS-C or remain asymptomatic after SARS-CoV-2 infection are yet to be established.

Cytokines and chemokines play a vital role in the initiation, prolongation, or downregulation of the immune response in COVID-19 in the pediatric population, including MIS-C [4]. It has been proposed that MIS-C, which shares comparable clinical features with Kawasaki disease, macrophage activation syndrome, and cytokine release syndrome, is the result of an aberrant immunological response to SARS-CoV-2 [5]. Children with MIS-C probably have elevated levels of cellular immunity and monocyte activation compared to mildly symptomatic children [6]. Cytokine levels may vary significantly among studies that use different proportions of age groups, timing of sampling, or diagnostic methods. Therefore, the exact activated immunological pathways that cause MIS-C after SARS-CoV-2 infection in children remain under investigation.

In contrast to the predominant T-helper 1 (Th1), IFN-mediated inflammatory response in adults, children reveal a substantial Th2 and Th17 immune response following exposure to SARS-CoV-2 [7,8]. The role of T-cell responses in the pathogenesis of MIS-C is significant and involves the activation of CD4^+^ and CD8^+^ lymphocytes, as well as the production of cytokines by lymphocytes [4]. CD8^+^ T cells in MIS-C exhibit greater cytotoxic signatures compared to those in healthy children [9].

The identification of specific immunophenotypic markers and their association with the clinical and epidemiological characteristics of pediatric patients could result in early recognition of severe disease or prolonged complications in different groups of patients and suggest which patients could benefit from targeted therapeutic agents.

The aim of this study is to prospectively compare the T-cell cytokine expression profile between children with convalescent COVID-19 or MIS-C.

## 2. Materials and Methods

### 2.1. Study Design and Participants

This was a prospective study involving children aged <16 years who were hospitalized at the “Aghia Sophia’’ Children’s Hospital, a 750-bed tertiary pediatric hospital, which is a reference center for pediatric COVID-19 cases between 1 January 2021 and 31 December 2022.

The study population included healthy, unvaccinated children, children with uncomplicated symptomatic SARS-CoV-2 infection (confirmed diagnosis by SARS-CoV-2 reverse-transcription polymerase chain reaction assay during the acute phase of disease) who had required hospitalization, as well as children who fulfilled the WHO or CDC criteria for MIS-C associated with COVID-19. The WHO criteria for MIS-C diagnosis include the following: age 0–19 years, fever for >3 days, and clinical signs of multisystem involvement (at least two of the following): rash, bilateral non-purulent conjunctivitis, or mucocutaneous inflammation signs (oral, hands, or feet), hypotension or shock, cardiac dysfunction, pericarditis, valvulitis, or coronary abnormalities (including echocardiographic findings or elevated troponin/BNP), evidence of coagulopathy (prolonged prothrombin time or partial prothrombin time, elevated D-dimer), acute gastrointestinal symptoms (diarrhea, vomiting, or abdominal pain), elevated markers of inflammation (e.g., erythrocyte sedimentation rate, C-reactive protein, or procalcitonin), no other obvious microbial cause of inflammation, including bacterial sepsis and staphylococcal/streptococcal toxic shock syndromes, and evidence of SARS-CoV-2 infection [2]. The CDC criteria for MIS-C diagnosis include the following: age <21 years, documented fever ≥38.0 °C (≥100.4 °F) or a report of subjective fever, severe illness requiring hospitalization or resulting in death, laboratory evidence of systemic inflammation (C-reactive protein ≥ 3 mg/dL), multisystem involvement (two or more of the following: shock, cardiovascular (elevated troponin or left ventricular ejection fraction <55% or coronary artery dilation, aneurysm, or ectasia on echocardiogram), hematologic (platelet count <150,000 cells/μL or absolute lymphocyte count <1000 cells/μL), gastrointestinal (abdominal pain, vomiting, or diarrhea), dermatologic (erythema or edema of hands or feet, oral mucositis, drying or fissuring of the lips, strawberry tongue, conjunctivitis, or other rash), confirmation of SARS-CoV-2 infection, and no alternative plausible diagnoses [10].

The study population was separated in four groups. The first group included eight children with uncomplicated COVID-19 in the convalescent phase who were recruited 30 days after the appearance of symptoms. The second group included four children with MIS-C during the acute phase of the syndrome (MISC_A). The third group included four children with MIS-C in the convalescent phase of the syndrome (MISC_C). In this group, blood samples were collected 30 days after the onset of the syndrome. In the healthy control group, four children who came for a well-child visit with negative SARS-CoV-2 anti-N IgG antibodies and no recent COVID-19 symptoms were included. Children diagnosed with immunocompromised conditions that could possibly affect humoral or cellular SARS-CoV-2 immune responses and children with history of SARS-CoV-2 immunization were excluded from the study.

### 2.2. Blood Sample Collection

Approximately 10 mL of whole peripheral blood was prospectively collected in potassium heparin syringes from all participants. Blood was drawn at one time point during patients’ hospitalization. In the MISC_A group, blood samples were collected before corticosteroids, intravenous immunoglobulin (IVIG), or other immunomodulatory medication administration. The isolation of peripheral blood mononuclear cells (PBMCs) was performed immediately after the collection of blood samples.

### 2.3. SARS-CoV-2 Antibody Detection

Serum samples for SARS-CoV-2 antibodies against SARS-CoV-2 nucleocapsid protein (anti-N) were tested in the Infectious Diseases Laboratory of the Choremeion Research Facility, First Department of Pediatrics, Medical School, National and Kapodistrian University of Athens, “Aghia Sophia” Children’s Hospital. Serum samples were analyzed using Elecsys^®^ Anti-SARS-CoV-2 (Roche Diagnostics, Basel, Switzerland) reagent on a Cobas e 411 immunoassay analyzer for the semiquantitative detection of total antibodies (IgA, IgM, and IgG), according to the manufacturer’s instructions.

### 2.4. Peripheral Blood Mononuclear Cell (PBMC) Isolation and Stimulation

Peripheral blood mononuclear cell (PBMC) isolation and stimulation were conducted in the Department of Immunology and Histocompatibility, “Aghia Sophia” Children’s Hospital, Athens, Greece.

PBMCs were isolated from heparinized whole blood by Ficoll gradient centrifugation [11] and were resuspended in Medium Complete culture media, i.e. Roswell Park Memorial Institute 1640 (RPMI 1640) media, supplemented with L-Glutamine (0.3 mg/mL), HEPES buffer (24 mM/mL), and 10% human Serum AB, instead of Fetal Bovine Serum, to minimize non-specific activation. The maximal final cell concentration was 10 × 10^7^ cells/L.

PBMCs were cultured either unstimulated (negative control) or in the presence of SARS-CoV-2 Spike antigenic peptide mix (Peptivator SARS-CoV-2 Prot_S proteater—Miltenyi Biotec, Bergisch Gladbach, Germany) at a final concentration of 1.67 nmol/mL. Each sample was incubated for a total of 17 h, in a 96-well plate, at 37 °C in a 5% CO_2_ atmosphere. A protein transport inhibitor (Brefeldin A, Invitrogen (Waltham, MA, USA), at a final concentration of 2 µg/mL) was added to the cell culture 30 min after the onset of incubation. Cell suspensions were finally collected and washed with an EDTA and albumin enriched buffer, to minimize non-specific monoclonal antibody binding.

### 2.5. Flow Cytometry and Statistical Analysis

SARS-CoV-2 Spike-specific T-cell subsets and their cytokine profile were assessed by flow cytometry in a single 13 color/15 marker combination as follows: (a) Surface staining for T-cell subset identification, i.e. CD3-APCAlexafluor750 (clone: UCHT1, Beckman Coulter, Miami, FL, USA), CD4-APCAlexafluor700 (clone: 13B8.2, Beckman Coulter), CD8-APC (clone: B9.11, Beckman Coulter), CD197-PE (clone: G043H7, Biolegend, San Diego, CA, USA), and CD45RA-ECD (clone: MEM-56, Exbio, Vestec, Czech Republic). (b) Intracellular cytokine and activation marker staining, i.e, IL-4-PC7 (clone: MP4-25D2, Beckman Coulter), IL-2-BV610 (clone: MQ1-17H12, Beckman Coulter), IL-17-PB (clone: BL168, Beckman Coulter), IFNγ-KRO (clone: 4S.B3, Beckman Coulter), TNF-α-BV660 (clone: Mab11, Beckman Coulter), CD137-BV750 (clone: 4B4-1, Biolegend), and Granzyme B-FITC (clone: QA16A02 Biolegend). (c) Monocyte, B cell and dead/apoptotic cell exclusion using CD14-PC5.5 (clone: RMO52, Beckman Coulter), CD19-PC5.5 (clone: J3-119, Beckman Coulter), and 7AAD.

The stained cell preparations were immediately analyzed in a 3 LASER, 13 color flow cytometer (DX Flex, Beckman Coulter, Miami, FL, USA), acquiring at least 1 million events per tube. The analysis of flow cytometry data was performed using Kaluza Analysis Software, version 2.1 (Beckman Coulter, Miami, FL, USA). The gating strategy is presented in Supplementary Figures S1–S3. The results were expressed as cells/million CD3^+^ and were calculated by the proportionate reduction of the respective unspecific events of the unstimulated sample. Non-parametric tests (Kruskal Wallis and Wilcoxon) were implemented for statistical analysis, using the SAS software (V9.4, SAS Institute Inc. https://www.sas.com). The graphs were created using GraphPad Prism 10 (San Diego, CA, USA).

### 2.6. Ethics Approval

The study protocol was approved by the Scientific and Bioethics Committee of the “Aghia Sophia” Children’s Hospital (No. 25609, 25 November 2020) according to the Declaration of Helsinki of 1964 and its subsequent amendments or comparable ethical standards. Written informed consent was obtained by the parents of all participants.

## 3. Results

### 3.1. Study Population

A total of 20 children with a median age (IQR) of 11.5 (7.25–14) years were included in the study with median age (IQR) 11.5 (7.25–14) years [males: 19/20 (95%), with no significant differences between the ages of four study groups]. The demographic characteristics and SARS-CoV-2 anti-N IgG titers of the study population are presented in Table 1.

The study population included children with 1. MIS-C during the acute phase of the disease (MISC_A) (*n* = 4), 2. MIS-C during the convalescent phase of the syndrome (one month after the acute phase) (MIS-C_C) (*n* = 4), 3. children in the convalescence phase of uncomplicated COVID-19 (one month after acute phase) (*n* = 8), and 4. healthy uninfected and unvaccinated children for SARS-CoV-2 (*n* = 4). The children of each group did not differ in age (*p*-value: 0.583) and therefore the study groups were age-matched. However, statistically significant differences were found in antibody levels between healthy children and the other three groups (*p*-value: 0.026).

Among the study participants, none of the children required intensive care unit hospitalization. All children with MIS-C or convalescent COVID-19 had detectable SARS-CoV-2 anti-N antibodies, indicating previous natural SARS-CoV-2 infection.

### 3.2. Flow Cytometry Analysis

Intracellular cytokine and activation marker expression data of SARS-CoV-2 Spike-specific T-cell subsets, as analyzed from children with MISC_A, MISC_C, post-COVID-19, and controls, are presented in Supplementary Table S1. The pairwise comparison analysis of the four clinical groups for each immunological parameter is presented in Supplementary Table S2. Significant differences are presented as follows.

From the combination of the six intracellular biomarkers and CD137 for CD4^+^, CD8^+^, CD4^+^CD8^+^, and CD4^−^CD8^−^, the biomarkers that were detected to distinguish MISC_A from MISC_C, post-COVID-19, and controls are as follows: CD4^+^IL-17^+^, CD8^+^IL-17^+^, CD8^+^IFNγ^+^, CD4^+^GRB^+^, CD8^+^CD137^+^, CD4^+^IL-2^+^, and CD8^+^IL-2^+^.

### 3.3. Immunological Differences Between MISC_A and Convalescent COVID-19 Children

To minimize bias from multiple comparisons across study groups, we applied the Bonferroni correction test for statistical analysis. After Bonferroni correction, analysis showed that 1. children with MISC-A had higher CD4^+^IL-17^+^ compared to controls (*p*-value: 0.081), and 2. children with MISC-A had higher CD8^+^IFN-γ^+^ compared to uncomplicated COVID-19 patients (*p*-value: 0.059).

### 3.4. IL17 Production by CD4^+^ and CD8^+^ T Cells

**Figure 1:**
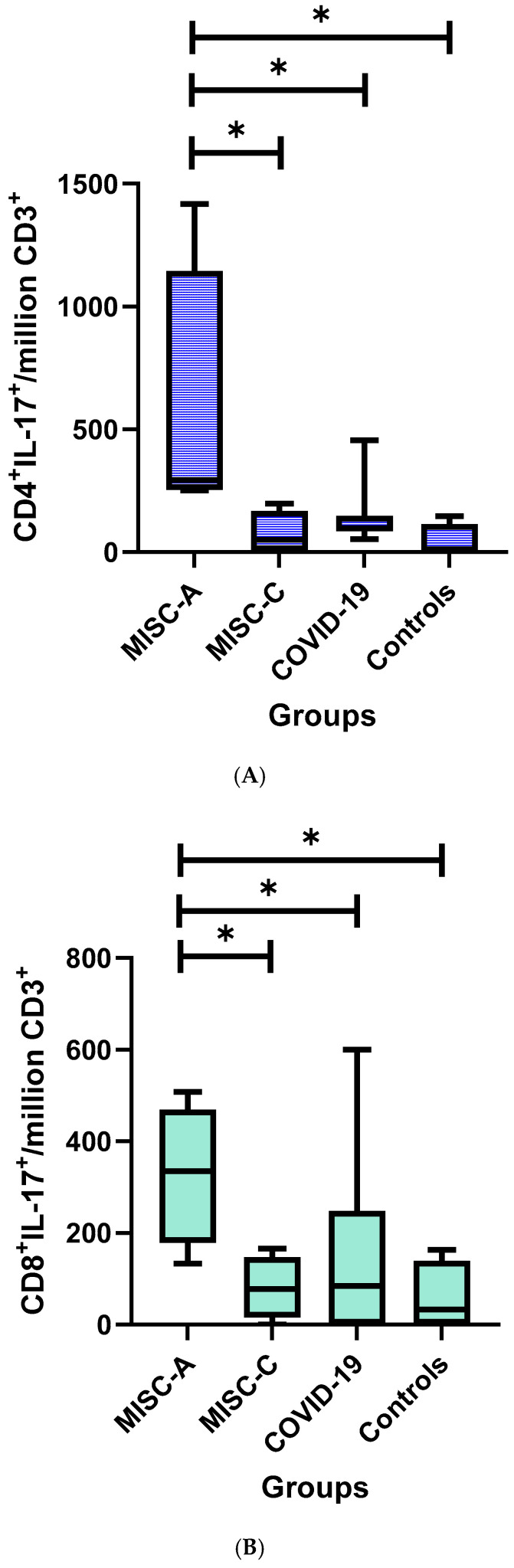

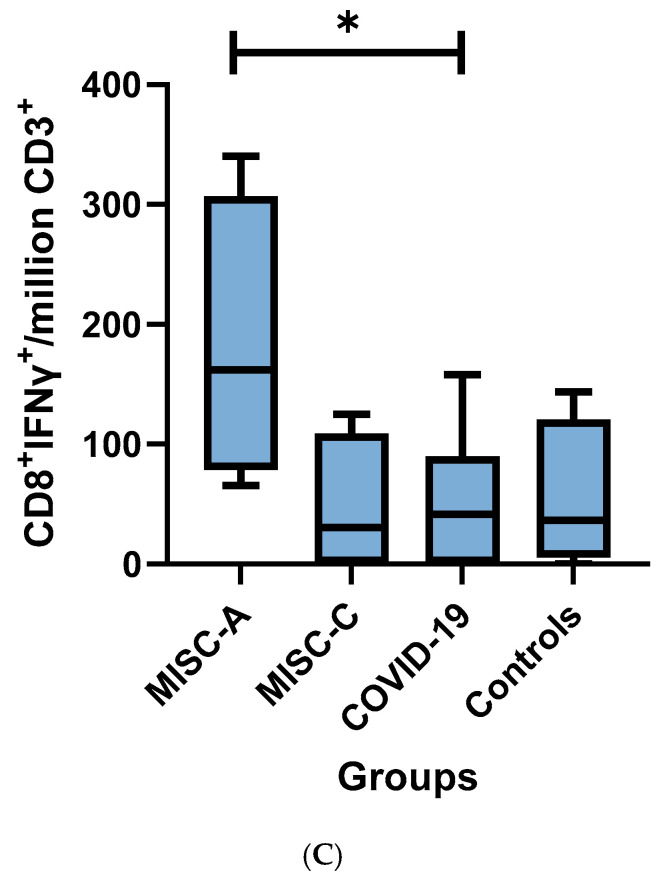
Boxplots of median (IQR) differences in 1. CD4^+^IL-17^+^/million CD3 values between the four study groups (**A**), 2. CD8^+^IL-17^+^/million CD3 values between the four study groups (**B**), 3. CD8^+^IFN-γ^+^/million CD3 values between the four study groups (**C**). *: *p*-value < 0.05.

From the comparison of the post-Spike antigen mix stimulation cytokine profile of MISC_A with the other three groups (MISC_C, post-COVID-19, and controls), significant differences were identified for CD4^+^IL-17^+^ and CD8^+^IL-17^+^ cells. The flow cytometry gating strategies for SARS-CoV-2 Spike-protein-specific PBMC expression of CD4^+^IL-17 and CD8^+^IL-17 in stimulated and unstimulated samples of each MISC_A, MISC_C, COVID-19, and control patient are presented in Supplementary Figure S1 and Supplementary Figure S2, respectively. The CD4^+^IL-17/million CD3^+^ median (IQR) values were higher in children with MISC_A compared to MISC_C (*p*-value: 0.03), COVID-19 (*p*-value: 0.03), and controls (*p*-value: 0.03) (Table S2) (Figure 1) (Supplementary Figure S1). The CD8^+^IL-17/million CD3^+^ values were higher in MISC_A compared to MISC_C, COVID-19, and controls (*p*-value: 0.05) (Supplementary Table S2) (Figure 1) (Supplementary Figure S2). Children with MISC_A had lower CD4^+^CD8^+^IL-17/million CD3^+^ median (IQR) values compared to healthy controls (*p*-value: 0.03) (Supplementary Table S2). No statistically significant differences were detected regarding pairwise comparison analysis of median (IQR) values for the four clinical groups for CD4^−^CD8^−^IL-17/million CD3^+^.

### 3.5. IFNγ Production by CD8^+^ T Cells

#### CD8^+^IFNγ^+^

The flow cytometry gating strategies for SARS-CoV-2 Spike-protein-specific PBMC expression of CD8^+^IFNγ in stimulated and unstimulated samples of each MISC_A, MISC_C, COVID-19, and control patient are presented in Supplementary Figure S3. The CD8^+^IFNγ^+^/million CD3^+^ values were higher in MISC_A compared to convalescent COVID-19 (*p*-value: 0.03) but were not significantly higher compared to MISC_C (*p*-value: 0.08) and compared to healthy controls (*p*-value: 0.08) (Supplementary Table S2) (Figure 1) (Supplementary Figure S3). MISC_A had significantly higher CD4^−^CD8^−^IFNγ^+^/million CD3^+^ median (IQR) values compared to controls (*p*-value: 0.03) (Supplementary Table S2). MISC_A had lower CD4^+^CD8^+^IFNγ^+^/million CD3^+^ median (IQR) values compared to COVID-19 (*p*-value: 0.02) and controls (*p*-value: 0.03) (Supplementary Table S2). No significant differences were detected regarding the pairwise analysis of the median (IQR) values for the MISC_A group compared with the other three clinical groups for CD4^+^IFNγ^+^/million CD3^+^, CD4^+^CD8^+^IFNγ^+^/million CD3^+^, or CD4^−^CD8^−^IFNγ^+^/million CD3^+^.

### 3.6. Other Significant Comparisons Between the Groups

#### 3.6.1. Granzyme B Production

MISC_A had significantly higher CD4^+^GRB^+^/million CD3^+^ values (*p*-value: 0.05) compared to healthy controls. The children with uncomplicated COVID-19 had higher CD4^+^GRB^+^/million CD3^+^ values (*p*-value: 0.02) compared to healthy controls (Supplementary Table S2). Compared to COVID-19, the children with MISC_C had lower CD4^+^GRB^+^/million CD3^+^ values (*p*-value: 0.02) (Supplementary Table S2). No significant differences were detected regarding pairwise comparison analysis of the median (IQR) values of the four clinical groups for CD8^+^GRB^+^/million CD3^+^, CD4^+^CD8^+^GRB^+^/million CD3^+^ or CD4^−^CD8^−^GRB^+^/million CD3^+^.

#### 3.6.2. CD8^+^ T-Cell Activation

The children with MISC_A had higher CD8^+^CD137^+^/million CD3^+^ values compared to MISC_C (*p*-value: 0.05) but the children with MISC_C had significantly lower CD8^+^CD137^+^/million CD3^+^ compared to healthy control group (*p*-value: 0.05) (Supplementary Table S2). The children with MISC_C had lower median (IQR) CD4^+^CD137^+^/million CD3^+^ values compared to healthy control group (*p*-value: 0.03) (Supplementary Table S2). MISC_A had higher CD4^−^CD8^−^CD137^+^/million CD3^+^ median (IQR) values compared to MISC_C (*p*-value: 0.05). No statistically significant differences were detected regarding pairwise comparison analysis of MISC_A with the other three clinical groups for CD4^+^CD8^+^CD137^+^/million CD3^+^ median (IQR) values.

#### 3.6.3. IL-2 Production by CD4^+^ and CD8^+^ T Cells

The children with MISC_A had higher CD4^+^IL-2^+^/million CD3^+^ values (*p*-value: 0.03) compared to convalescent COVID-19, higher CD8^+^IL-2^+^/million CD3^+^ (*p*-value: 0.05) compared to healthy controls, and higher CD4^−^CD8^−^IL-2^+^/million CD3^+^ median (IQR) values compared to MISC_C (*p*-value: 0.03) (Supplementary Table S2). No statistically significant differences were detected regarding pairwise comparison analysis of the four clinical groups for CD4^+^CD8^+^IL-2^+^/million CD3^+^ median (IQR) values.

## 4. Discussion

In this study, the cytokine expression profile in children with COVID-19 was investigated by using six different intracellular monoclonal antibody markers post-SARS-CoV-2 stimulation on multiple SARS-CoV-2 T-cell subsets defined by CD4 and CD8 and one activation marker (CD137) with flow cytometry.

Immune dysregulation in children with COVID-19 was investigated by using 15 different extracellular and intracellular monoclonal antibody markers with flow cytometry. Cytokines and chemokines play a vital role in the initiation, prolongation, or downregulation of the immune response in COVID-19 in the pediatric population, including MIS-C. This technique, which allows COVID-19 immune system decryption at single lymphocyte level, could be beneficial in an immunologically complicated condition such as MIS-C. The deciphering of cellular immune responses among age-matched children across the SARS-CoV-2 severity spectrum should shed light on the role of virus-specific T-cell immunity with the identification of T-cell subpopulations or cytokines that could constitute possible diagnostic immunological biomarkers. The use of freshly collected PBMCs has provided a high-quality material which is crucial for the interpretation of functional assays.

In the present study, it was shown that the T cells of children presenting with MIS-C one month after COVID-19 infection could be polarized towards two of the six cytokines studied: IL-17 and IFN-γ. These cytokine’s intracellular expression level post-Spike protein antigen mix stimulation could distinguish acute MIS-C from uncomplicated convalescent COVID-19 and could serve as possible immunological biomarkers for early MIS-C detection. However, this is a hypothesis that should be confirmed in other studies with a higher number of participants.

There are studies that investigate cytokine expression in MIS-C and involve lymphocyte-specific cytokine expression after PBMC isolation from fresh, whole peripheral blood. Most published studies predominantly use serum [12,13] or plasma [14,15] in order to evaluate cytokine expression. There is a study by Rybkina et al. based on fresh blood collection and PBMC isolation that also showed distinct cellular immune responses between MIS-C and uncomplicated COVID-19 patients, however the aim of the study was to identify certain transcriptional signature differences of TCR gene expression between MIS-C in the acute and convalescent phases [11]. Kumar et al. performed a study using fresh, non-freeze-thawed blood of MIS-C patients; however, no PBMC isolation was implemented [16]. It is important to interpret results regarding cytokines from non-specific inflamed tissues (serum, plasma, and whole blood) from other studies that specifically reflect the activation of CD4^+^ and CD8^+^, both of which play a key role in conditions characterized by immune dysregulation, such as MIS-C. Despite the limited number of participants, this study could possibly help distinguish possible cellular immune responses from certain lymphocyte subtypes.

IL-17 expressed by either CD4^+^ or CD8^+^ T cells possibly plays a key role in MIS-C pathogenesis, as children with acute MIS-C had higher IL-17 values compared to convalescent COVID-19 or MIS-C patients. Although it was carried out with a limited number of participants, the increasing trend in IL-17 in numerous PBMC populations in MIS-C improves the reliability of the finding. The increasing trend of CD8-producing IL-17 in patients with acute MIS-C in parallel with the decrease in convalescent MIS-C suggests that IL-17 may be involved in inflammatory mechanisms of the acute phase of SARS-CoV-2 infection. An aberrant increase of IL-17 in MIS-C compared to pediatric COVID-19 patients was also detected in the study by Gruber et al. in freeze-thawed PBMC samples by flow cytometry [17]. Serum IL-17 was detected at higher levels in MIS-C compared to COVID-19 or healthy controls [13,18]. Th17 cells release IL-17A which stimulates the production of proinflammatory cytokines such as IL-17, IL-22, and IL-26 and plays an important role in neutrophils recruitment as well as cardiovascular clinical manifestations [19]. IL-17 also has significant correlations with immune-mediated chronic inflammatory conditions, such as psoriasis, ankylosing spondylitis, rheumatoid arthritis, and Crohn’s disease [20]. The inflammatory mediator IL-17 also has a prominent role in Kawasaki disease progress [21] while Th17 cells appear to have a key role in promoting chronic inflammatory responses in chronic infection, allergies, and autoimmunity [22].

Children with acute MIS-C tend to have elevated CD8^+^IFNγ values compared to the other study groups, especially compared to convalescent COVID-19 patients, indicating the involvement of IFNγ in the mechanisms of acute phase MIS-C. The activation of IFNγ immunological pathways in MIS-C has also been documented in other studies in which cytokine detection was performed either in serum or in whole blood [17,18,23]. In the study by Gruber et al., IFN-gamma-producing T cells increased significantly in children with MIS-C compared to children with acute COVID-19 and healthy controls [17]. The potential significance of the IFN pathway in a select subset of children with COVID-19 may also suggest an inherent genetic susceptibility, such as the presence of polymorphisms or specific mutations. According to a study conducted by Zhang et al., patients who have the potential to develop fatal COVID-19 have been found to display rare modifications in 13 loci that result in loss of function and, consequently, an abnormal IFN pathway activation [24].

In this study, CD8^+^CD137^+^ data in convalescent MIS-C patients supporting immunological differences with acute MIS-C, pediatric convalescent COVID-19, and healthy controls were reported. CD137^+^ as a potential biomarker of disease severity has already been investigated in adult COVID-19 patients [25]. Acute MIS-C had higher levels of CD8^+^CD137^+^ compared to convalescent MIS-C and, interestingly, the values were approximately zero in convalescent MIS-C. This could possibly indicate CD8^+^CD137^+^ T-cell exhaustion and apoptosis one month after the onset of the syndrome, a condition also mentioned in other studies [26,27]. Ramaswamy et al. also highlighted the reduction of antigenic presentation molecules in moderate and severe acute MIS-C [28]. Normal T-cell recognition involves an antigen being taken up by an antigen-presenting cell, processed, expressed on the cell surface in conjunction with class II MHC, and recognized by an antigen-specific T-cell receptor [29]. Antigen-presenting cells play a primary role in host immune responses especially in superantigens, which interact directly with the invariant region of the class II MHC molecule, bypassing the antigen-presenting cell process [29]. This process has been clearly documented for *S. aureus*-associated Toxic Shock Syndrome (TSS), in which staphylococcal enterotoxins behave as superantigens capable of activating large numbers of T cells and leading to significant cytokine production. The SARS-CoV-2 viral spike (S) protein may act as a superantigen, triggering a cytokine storm that leads to the development of MIS-C, a condition with resemblances to TSS [30]. Superantigen-mediated T-cell activation was identified as being linked with the HLA class I alleles A02, B35, and C04 in MIS-C patients through the TRBV11-2 (V21.3) skewing of the TCR repertoire in a group of CDR3-independent individuals. The superantigen-like motif of the SARS-CoV-2 S glycoprotein was found to correlate significantly with polyacidic residues in the V chain encoded by TRBV11-2 (V21.3), suggesting that SARS-CoV-2 S protein may directly mediate the extension of TRBV11-2 [9]. However, during the acute phase of inflammation, SARS-CoV-2 is usually undetectable in patients with MIS-C, meaning that the connection between superantigen theory and MIS-C has yet to be proven.

Children with COVID-19, including MIS-C in the acute and convalescent phases of the disease, were shown to have lower proportions of lymphocyte-specific antigen-specific cells (CD137^+^) compared to healthy controls. This is consistent with the findings of Singh et al. which showed lower T-cell and SARS-CoV-2 antigen-specific T cells in 20 MIS-C patients compared to convalescent COVID-19 and healthy controls in plasma and cryopreserved PMBCs that were thawed overnight [26]. In a study by Lam et al. in which PBMC isolation was performed in liquid nitrogen, a robust expression of antigen-specific cells in 24 MIS-C compared to 10 uncomplicated COVID-19 patients was observed [31]. Higher antigen-specific T-cell responses in 24 MIS-C patients compared to 10 uncomplicated COVID-19 patients were also observed in the study of Conway et al., however a 10-day instead of a 2-day protocol for PBMC isolation was used [32]. These differences could not only possibly be attributed to CD8^+^ exhaustion and apoptosis but also to different PBMC isolation techniques.

Despite the limited number of participants, SARS-CoV-2-specific CD4^+^GRB^+^ values were found to be distinct among MIS-C, COVID-19, and healthy controls and were specifically lower in acute MIS-C compared to COVID-19 but higher compared to healthy controls. It is possible that robust memory T-cell activation during the MIS-C acute phase is resolved during convalescence, possibly due to T-cell exhaustion and apoptosis, and this has also been supported by Carter et al. who evaluated immune responses in the same MIS-C patients during the acute and convalescent phases [33].

As a biomarker of cytotoxicity, Granzyme B has been found to induce inflammation by enhancing cytokine release [34]. Granzyme B is released by regulatory T cells (tregs) to eliminate CD4^+^ T cells that have not been exposed to restricted-to-peripheral-tissue host cells, hence preventing an autoimmune response to selfantigens [34]. Serum Granzyme B production has been found to be enhanced in MIS-C patients compared to uncomplicated COVID-19 [35] and in MIS-C patients without myocarditis compared to MIS-C with myocardiac involvement [36]. This contradicts with the findings of our study, possibly due to the limited number of participants and the different laboratory techniques, since serum cytokines fail to specifically distinguish T-cell-produced cytokines.

The detection of certain biomarkers in this study, mainly IL-17 and IFNγ, distinguish MISC_A from other COVID-19 patients and could assist in various clinical applications and treatment options. The implication of multiple assays that involve IL-17 and IFNγ detection in COVID-19 patients could distinguish those who could develop MIS-C in the future. The increase of IL-17 in MISC_A patients could raise awareness for more future studies on *novel* diagnostic assays and treatment strategies involving IL-17-targeted biologic therapies, such as Secukinumab, Bimekizumab, Brodamulab, and Ixekizumab, which have already effectively been used in other IL-17-mediated conditions, such as Crohn’s disease, psoriasis, and ankylosing spondylitis [37]. In another study by our group, QuantiFERON SARS-CoV-2 assay, a fast and effective IFNγ release assay used to detect IFN-γ production from CD8^+^ T cells, proved effective in children with COVID-19 [38]. Based on the results of the current study, this assay could also be helpful in MIS-C early detection and treatment.

Our study has specific limitations including the low number of participants as well as the male predominance in the study groups. Nevertheless, this is a study which used a large number of immunological parameters and included only SARS-CoV-2 unvaccinated children, thus interpreting cellular immune responses only from SARS-CoV-2 natural infection and excluding the interference of T-cell stimulation as a result of SARS-CoV-2 vaccination. Through the prospective use of fresh, non-freeze-thawed, whole peripheral blood collection, alongside the absence of pharmaceutic immunosuppression of patients, the risk of cellular death and any pharmaceutic interference with the cytokine expression profile was aimed to be diminished.

## 5. Conclusions

The deciphering of specific cellular immune responses and the identification of diagnostic immunological biomarkers in children with MIS-C is important for early diagnosis and therapeutic intervention. Children with MIS-C after acute COVID-19 infection were identified with highly active T cells for IL-17 and IFNγ production compared to those with uncomplicated convalescent COVID-19. These results are supportive of an indication that IL-17 and IFNγ could possibly be used for the laboratory diagnosis of MIS-C. Further studies with an increased number of participants and the verification with proteomics and novel computational tools could identify easily used biomarkers for the early detection of MIS-C.

## Figures and Tables

**Table 1:** Demographic characteristics and SARS-CoV-2 anti-N IgG titers of the study population (*n* = 20). Age and SARS-CoV-2 anti-N IgG antibody values are presented as median and interquartile range. Statistically significant differences (*p*-value ≤ 0.05) are marked in bold.

Group	Age (Years)	SARS-CoV-2 Anti-N IgG (COI)
MISC_A (*n* = 4)	13.0 (8.0–15.0)	42.8 (12.3–131.9)
MISC_C (*n* = 4)	9.5 (5.8–12.5)	26.4 (21.2–93.2)
COVID-19 (*n* = 8)	12.5 (6.6–14.0)	32.8 (14.9–75.0)
Control (*n* = 4)	11.1 (7.4–14.0)	0.07 (0.07–0.09)
*p*-value	0.583	**0.026**

## Data Availability

The data that support the findings of this study are available from the corresponding author upon reasonable request.

## Supplementary Materials

The following supporting information can be downloaded at: https://www.mdpi.com/article/10.3390/children11111278/s1, Figure S1: Flow cytometry gating strategy for SARS-CoV-2 Spike-protein-specific PBMC expression of CD4^+^IL-17 in stimulated (left) and unstimulated (right) samples of each MISC_A (Panel A), MISC_C (Panel B), COVID-19 (Panel C) and controls (Panel D). Blue colors represent CD4^+^ T-cells and pink colors represent CD4^+^CD137^+^ T-cells.; Figure S2: Flow cytometry gating strategy for SARS-CoV-2 Spike-protein-specific PBMC expression of CD8^+^IL-17 in stimulated (left) and unstimulated (right) samples of each MISC_A (Panel A), MISC_C (Panel B), COVID-19 (Panel C) and controls (Panel D); Figure S3: Flow cytometry gating strategy for SARS-CoV-2 Spike-protein-specific PBMC expression of CD8^+^IFN-γ in stimulated (left) and unstimulated (right) samples of each MISC_A (Panel A), MISC_C (Panel B), COVID-19 (Panel C) and controls (Panel D); Table S1: Flow cytometry values regarding the combination of the six intracellular biomarkers and CD137 for CD4, CD8, CD4^+^CD8^+^ and CD4^−^CD8^−^ that were analyzed from children with MISC_A, MISC_C, post-COVID-19, and controls. Statistically significant differences (*p*-value ≤ 0.05) are marked in bold. Table S2: Pairwise comparison analysis of the four clinical groups (MISC_A, MISC_C, COVID-19 and control group) for combination of the six intracellular biomarkers and CD137 for CD4, CD8, CD4^+^CD8^+^ and CD4^−^CD8^−^. Statistically significant differences (*p*-value ≤ 0.05) are marked in bold.

## References

[1] Feldstein L.R., Rose E.B., Horwitz S.M., Collins J.P., Newhams M.M., Son M.B.F., Newburger J.W., Kleinman L.C., Heidemann S.M., Martin A.A. (2020). Multisystem Inflammatory Syndrome in U.S. Children and Adolescents. N. Engl. J. Med..

[2] Multisystem Inflammatory Syndrome in Children and Adolescents Temporally Related to COVID-19. https://www.who.int/news-room/commentaries/detail/multisystem-inflammatory-syndrome-in-children-and-adolescents-with-covid-19.

[3] HAN Archive—00432|Health Alert Network (HAN). https://www.cdc.gov/mis/hcp/case-definition-reporting/index.html.

[4] Filippatos F., Tatsi E.-B., Michos A. (2023). Immunology of Multisystem Inflammatory Syndrome after COVID-19 in Children: A Review of the Current Evidence. Int. J. Mol. Sci..

[5] Lee P.Y., Day-Lewis M., Henderson L.A., Friedman K.G., Lo J., Roberts J.E., Lo M.S., Platt C.D., Chou J., Hoyt K.J. (2020). Distinct clinical and immunological features of SARS-CoV-2-induced multisystem inflammatory syndrome in children. J. Clin. Investig..

[6] Bartsch Y.C., Wang C., Zohar T., Fischinger S., Atyeo C., Burke J.S., Kang J., Edlow A.G., Fasano A., Baden L.R. (2021). Humoral signatures of protective and pathological SARS-CoV-2 infection in children. Nat. Med..

[7] Jia R., Wang X., Liu P., Liang X., Ge Y., Tian H., Chang H., Zhou H., Zeng M., Xu J. (2020). Mild Cytokine Elevation, Moderate CD4^+^ T Cell Response and Abundant Antibody Production in Children with COVID-19. Virol. Sin..

[8] Pierce C.A., Preston-Hurlburt P., Dai Y., Aschner C.B., Cheshenko N., Galen B., Garforth S.J., Herrera N.G., Jangra R.K., Morano N.C. (2020). Immune responses to SARS-CoV-2 infection in hospitalized pediatric and adult patients. Sci. Transl. Med..

[9] Porritt R.A., Paschold L., Rivas M.N., Cheng M.H., Yonker L.M., Chandnani H., Lopez M., Simnica D., Schultheiß C., Santiskulvong C. (2021). HLA class I–associated expansion of TRBV11-2 T cells in multisystem inflammatory syndrome in children. J. Clin. Investig..

[10] Information for Healthcare Providers About Multisystem Inflammatory Syndrome in Children (MIS-C)|CDC. https://archive.cdc.gov/www_cdc_gov/mis/mis-c/hcp/index.html.

[11] Rybkina K., Bell J.N., Bradley M.C., Wohlbold T., Scafuro M., Meng W., Korenberg R.C., Davis-Porada J., Anderson B.R., Weller R.J. (2023). SARS-CoV-2 infection and recovery in children: Distinct T cell responses in MIS-C compared to COVID-19. J. Exp. Med..

[12] Ahmed Mostafa G., Mohamed Ibrahim H., Al Sayed Shehab A., Mohamed Magdy S., AboAbdoun Soliman N., Fathy El-Sherif D. (2022). Up-regulated serum levels of interleukin (IL)-17A and IL-22 in Egyptian pediatric patients with COVID-19 and MIS-C: Relation to the disease outcome. Cytokine.

[13] Gelzo M., Castaldo A., Giannattasio A., Scalia G., Raia M., Esposito M.V., Maglione M., Muzzica S., D’anna C., Grieco M. (2022). MIS-C: A COVID-19-as sociated condition between hypoimmunity and hyperimmunity. Front. Immunol..

[14] Diaz F., Bustos B.R., Yagnam F., Karsies T.J., Vásquez-Hoyos P., Jaramillo-Bustamante J.C., Gonzalez-Dambrauskas S., Drago M., Cruces P. (2021). Comparison of Interleukin-6 Plasma Concentration in Multisystem Inflammatory Syndrome in Children Associated with SARS-CoV-2 and Pediatric Sepsis. Front. Pediatr..

[15] Diorio C., McNerney K.O., Lambert M., Paessler M., Anderson E.M., Henrickson S.E., Chase J., Liebling E.J., Burudpakdee C., Lee J.H. (2020). Evidence of thrombotic microangiopathy in children with SARS-CoV-2 across the spectrum of clinical presentations. Blood Adv..

[16] Kumar N.P., Venkataraman A., Nancy A., Moideen K., Varadarjan P., Selladurai E., Sangaralingam T., Selvam R., Thimmaiah A., Natarajan S. (2022). Enhanced Severe Acute Respiratory Syndrome Coronavirus 2 Antigen–Specific Systemic Immune Responses in Multisystem Inflammatory Syndrome in Children and Reversal After Recovery. J. Infect. Dis..

[17] Gruber C.N., Patel R.S., Trachtman R., Lepow L., Amanat F., Krammer F., Wilson K.M., Onel K., Geanon D., Tuballes K. (2023). Mapping Systemic Inflammation and Antibody Responses in Multisystem Inflammatory Syndrome in Children (MIS-C). Cell.

[18] Gurlevik S.L., Ozsurekci Y., Sağ E., Oygar P.D., Kesici S., Akca K., Cuceoglu M.K., Basaran O., Göncü S., Karakaya J. (2022). The difference of the inflammatory milieu in MIS-C and severe COVID-19. Pediatr. Res..

[19] Bettelli E., Korn T., Oukka M., Kuchroo V.K. (2008). Induction and effector functions of T(H)17 cells. Nature.

[20] Ruiz de Morales J.M.G., Puig L., Daudén E., Cañete J.D., Pablos J.L., Martín A.O., Juanatey C.G., Adán A., Montalbán X., Borruel N. (2020). Critical role of interleukin (IL)-17 in inflammatory and immune disorders: An updated review of the evidence focusing in controversies. Autoimmun. Rev..

[21] Consiglio C.R., Cotugno N., Sardh F., Pou C., Amodio D., Rodriguez L., Tan Z., Zicari S., Ruggiero A., Pascucci G.R. (2020). The Immunology of Multisystem Inflammatory Syndrome in Children with COVID-19. Cell.

[22] Mills K.H.G. (2023). IL-17 and IL-17-producing cells in protection versus pathology. Nat. Rev. Immunol..

[23] Lapp S.A., Abrams J., Lu A.T., Hussaini L., Kao C.M., Hunstad D.A., Rosenberg R.B., Zafferani M.J., Ede K.C., Ballan W. (2022). Serologic and Cytokine Signatures in Children with Multisystem Inflammatory Syndrome and Coronavirus Disease 2019. Open Forum Infect. Dis..

[24] Zhang Q., Bastard P., Liu Z., Le Pen J., Moncada-Velez M., Chen J., Ogishi M., Sabli I.K.D., Hodeib S., Korol C. (2020). Inborn errors of type I IFN immunity in patients with life-threatening COVID-19. Science.

[25] Marques M.d.O., Abdo A., Silva P.B., Junior A.S., Alves L.B.d.O., Costa J.V.G., Martin J., Bachour P., Baiocchi O.C.G. (2022). Soluble CD137 as a potential biomarker for severe COVID-19. Immunol. Lett..

[26] Singh V., Obregon-Perko V., Lapp S.A., Horner A.M., Brooks A., Macoy L., Hussaini L., Lu A., Gibson T., Silvestri G. (2022). Limited induction of SARS-CoV-2-specific T cell responses in children with multisystem inflammatory syndrome compared with COVID-19. JCI Insight.

[27] Bhuiyan T.R., Al Banna H., Kaisar M.H., Karmakar P.C., Hakim A., Akter A., Ahmed T., Tauheed I., Islam S., Hasnat M.A. (2022). Correlation of antigen-specific immune response with disease severity among COVID-19 patients in Bangladesh. Front. Immunol..

[28] Ramaswamy A., Brodsky N.N., Sumida T.S., Comi M., Asashima H., Hoehn K.B., Li N., Liu Y., Shah A., Ravindra N.G. (2021). Immune dysregulation and autoreactivity correlate with disease severity in SARS-CoV-2-associated multisystem inflammatory syndrome in children. Immunity.

[29] Schlievert P.M. (1993). Role of superantigens in human disease. J. Infect. Dis..

[30] Noval Rivas M., Porritt R.A., Cheng M.H., Bahar I., Arditi M. (2021). COVID-19-associated multisystem inflammatory syndrome in children (MIS-C): A novel disease that mimics toxic shock syndrome-the superantigen hypothesis. J. Allergy Clin. Immunol..

[31] Lam K.P., Chiñas M., Julé A.M., Taylor M., Ohashi M., Benamar M., Crestani E., Son M.B.F., Chou J., Gebhart C. (2022). SARS-CoV-2-specific T cell responses in patients with multisystem inflammatory syndrome in children. Clin. Immunol..

[32] Conway S.R., Lazarski C.A., Field N.E., Jensen-Wachspress M., Lang H., Kankate V., Durkee-Shock J., Kinoshita H., Suslovic W., Webber K. (2022). SARS-CoV-2-Specific T Cell Responses Are Stronger in Children with Multisystem Inflammatory Syndrome Compared to Children with Uncomplicated SARS-CoV-2 Infection. Front. Immunol..

[33] Carter M.J., Fish M., Jennings A., Doores K.J., Wellman P., Seow J., Acors S., Graham C., Timms E., Kenny J. (2020). Peripheral immunophenotypes in children with multisystem inflammatory syndrome associated with SARS-CoV-2 infection. Nat. Med..

[34] Afonina I.S., Cullen S.P., Martin S.J. (2010). Cytotoxic and non-cytotoxic roles of the CTL/NK protease granzyme B. Immunol. Rev..

[35] Venkataraman A., Kumar N.P., Hanna L.E., Putlibai S., Karthick M., Rajamanikam A., Sadasivam K., Sundaram B., Babu S. (2021). Plasma biomarker profiling of PIMS-TS, COVID-19 and SARS-CoV2 seropositive children—A cross-sectional observational study from southern India. eBioMedicine.

[36] de Cevins C., Luka M., Smith N., Meynier S., Magérus A., Carbone F., García-Paredes V., Barnabei L., Batignes M., Boullé A. (2021). A monocyte/dendritic cell molecular signature of SARS-CoV-2-related multisystem inflammatory syndrome in children with severe myocarditis. Med.

[37] Țiburcă L., Bembea M., Zaha D.C., Jurca A.D., Vesa C.M., Rațiu I.A., Jurca C.M. (2022). The Treatment with Interleukin 17 Inhibitors and Immune-Mediated Inflammatory Diseases. Curr. Issues Mol. Biol..

[38] Dourdouna M.M., Tatsi E.B., Syriopoulou V., Michos A. (2023). Evaluation of T cell responses with the QuantiFERON SARS-CoV-2 assay in individuals with 3 doses of BNT162b2 vaccine, SARS-CoV-2 infection, or hybrid immunity. Diagn. Microbiol. Infect. Dis..

