# Supplementary material for: Comparison οf Immune Responses Through Multiparametric T-Cell Cytokine Expression Profile Between Children with Convalescent COVID-19 or Multisystem Inflammatory Syndrome
Source: Children (Basel). 2024 Oct 23;11(11):1278. doi: 10.3390/children11111278 (PMC11592800; doi:10.3390/children11111278)
Supplement: Supplementary file 1 [file children-11-01278-s001.zip › children-3259896-supplementary.pdf]

Supplementary Table S1. Flow cytometry values regarding the combination of the 6 intracellular biomarkers and CD137 for CD4, CD8, $CD4^{+}CD8^{+}$ and $CD4^{-}CD8^{-}$ that were analyzed from children with MISC_A, MISC_C, post-COVID-19 and controls. Statistically significant differences (*P*-value≤0.05) are marked in bold.

		MISC_A	MISC_C	COVID-19	Control	*P*-value
**$CD3^+CD4^+$**	**$CD3^+CD4^+CD137^+$ /million $CD3^+$**	24.6 (7.6-523.8)	6.4 (0.0-68.0)	0.0 (0.0-357.0)	492.7 (358.6-593.1)	0.314
	**$CD3^+CD4^+GRB^+$/million $CD3^+$**	1990.8 (1383.9-4411.5)	856.0 (340.8-1345.7)	2787.2 (2446.8-5833.3)	76.4 (0.0-561.5)	**0.009**
	**$CD3^+CD4^+IFN\gamma^+$/million $CD3^+$**	97.6 (41.0-292.0)	56.2 (41.3-87.7)	41.5 (5.4-186.2)	30.0 (0.0-72.9)	0.549
	**$CD3^+CD4^+IL17^+$/million $CD3^+$**	293.0 (256.4-870.9)	50.7 (8.4-140.5)	96.7 (89.2-135.4)	8.7 (0.0-82.4)	**0.017**
	**$CD3^+CD4^+IL2^+$/million $CD3^+$**	217.0 (178.3-291.9)	167.4 (84.2-193.2)	33.5 (6.5-102.8)	40.2 (14.8-220.1)	0.159
	**$CD3^+CD4^+IL4^+$/million $CD3^+$**	18.9 (7.6-44.6)	0.0 (0.0-0.0)	0.0 (0.0-24.2)	10.7 (2.6-32.9)	0.37
	**$CD3^+CD4^+TNF\text{-}\alpha^+$/million $CD3^+$**	145.0 (55.8-219.7)	140.4 (78.4-185.2)	75.8 (17.0-198.7)	53.5 (3.2-111.9)	0.656
**$CD3^+CD8^+$**	**$CD3^+CD8^+CD137^+$/million $CD3^+$**	71.6 (15.3-473-6)	0.0 (0.0-0.0)	0.0 (0.0-159.1)	96.5 (15.0-253.7)	0.178
	**$CD3^+CD8^+GRB^+$/million $CD3^+$**	8908.0 (5559.3-26271.0)	4752.3 (3719.7-17558.8)	25007.1 (14183.1-35634.3)	5836.4 (0.0-44256.7)	0.455
	**$CD3^+CD8^+IFN\gamma^+$/million $CD3^+$**	162.2 (91.6-273.4)	30.3 (0.0-92.8)	41.5 (0.0-77.4)	36.6 (11.2-97.3)	0.124
	**$CD3^+CD8^+IL17^+$/million $CD3^+$**	335.2 (225.8-429.9)	78.0 (31.9-128.9)	84.1 (0.0-204.6)	33.2 (0.0-114.6)	0.1

	**CD3$^+$CD8$^+$IL2$^+$/million CD3$^+$**	99.2 (62.1-103.6)	61.0 (28.6-73.9)	35.9 (19.9-61.5)	12.4 (4.4-23.0)	0.1
	**CD3$^+$CD8$^+$IL4$^+$/million CD3$^+$**	8.9 (0.0-23.8)	4.1 (1.0-32.9)	0.5 (0.0-23.1)	7.2 (0.0-22.4)	0.948
	**CD3$^+$CD8$^+$TNF-α$^+$/million CD3$^+$**	50.7 (19.9-113.6)	65.7 (62.2-74.4)	20.7 (5.4-63.3)	0.0 (0.0-61.2)	0.329
**CD3$^+$CD4$^-$CD8$^-$**	**CD3$^+$CD4$^-$CD8$^-$CD137$^+$/million CD3$^+$**	145.0 (51.4-274.0)	0.0 (0.0-0.0)	0.0 (0.0-33.5)	310.4 (180.5-492.9)	**0.021**
	**CD3$^+$CD4$^-$CD8$^-$GRB$^+$/million CD3$^+$**	13742.7 (9692.3-20500.2)	5531.9 (3473.8-29238.1)	20176.3 (11304.6-31790.1)	12890.8 (7382.9-29996.6)	0.4
	**CD3$^+$CD4$^-$CD8$^-$IFNγ$^+$/million CD3$^+$**	168.4 (104.7-233.8)	32.7 (14.4-133.2)	56.7 (10.5-219.5)	12.3 (0.2-35.2)	0.245
	**CD3$^+$CD4$^-$CD8$^-$IL17$^+$/million CD3$^+$**	99.8 (36.3-238.2)	54.7 (16.6-133.6)	35.5 (0.0-205.9)	111.4 (37.0-186.7)	0.960
	**CD3$^+$CD4$^-$CD8$^-$IL2$^+$/million CD3$^+$**	146.9 (84.3-164.5)	25.3 (9.5-34.0)	35.7 (14.6-204.0)	50.8 (0.0-103.1)	0.181

	**$CD3^+CD4^-CD8^-IL4^+$/million $CD3^+$**	12.6 (0.0-54.3)	32.3 (10.9-55.9)	0.0 (0.0-17.1)	16.1 (0.0-36.9)	0.253
	**$CD3^+CD4^-CD8^-TNF\text{-}\alpha^+$/million $CD3^+$**	69.3 (47.8-148.1)	29.4 (5.7-56.5)	8.3 (0.0-139.6)	0.4 (0.0-125.7)	0.395
**$CD3^+CD4^+CD8^+$**	**$CD3^+CD4^+CD8^+CD137^+$/million $CD3^+$**	3.5 (0.2-24.4)	0.0 (0.0-3.6)	2.5 (0.0-21.0)	30.0 (24.8-33.3)	0.154
	**$CD3^+CD4^+CD8^+GRB^+$/million $CD3^+$**	29.7 (10.8-311.3)	138.8 (67.8-168.2)	1.4 (0.0-93.4)	75.1 (26.3-115.9)	0.531
	**$CD3^+CD4^+CD8^+IFN\gamma^+$/million $CD3^+$**	0.0 (0.0-0.0)	5.1 (0.0-18.2)	3.8 (1.3-27.1)	23.6 (7.1-70.1)	**0.034**
	**$CD3^+CD4^+CD8^+IL17^+$/million $CD3^+$**	0.0 (0.0-0.0)	0.0 (0.0-14.1)	0.2 (0.0-38.6)	20.2 (10.1-29.4)	0.057
	**$CD3^+CD4^+CD8^+IL2^+$/million $CD3^+$**	5.8 (1.7-26.8)	9.2 (7.6-11.4)	13.3 (2.4-24.5)	20.3 (8.2-48.7)	0.688
	**$CD3^+CD4^+CD8^+IL4^+$/million $CD3^+$**	11.7 (5.8-17.7)	3.8 (0.0-8.0)	0.0 (0.0-6.0)	2.4 (0.0-11.7)	0.286

	**CD3$^+$CD4$^+$CD8$^+$TNF-α$^+$/million CD3$^+$**	22.9 (7.6-40.6)	8.2 (5.1-17.5)	2.4 (1.0-12.1)	1.5 (0.4-16.6)	0.168

Abbreviations: MISC_A: Multisystem Inflammatory Syndrome in Children in acute phase of the syndrome; MISC_C: Multisystem Inflammatory Syndrome in Children in the convalescent phase of the syndrome; GRB: Granzyme B; IL17: Interleukin 17; IFNγ: Interferon-gamma; IL2: Interleukin 2; IL4: Interleukin 4; TNF-A: Tumor Necrosis Factor- α.

Supplementary Table S2. Pairwise comparison analysis of the four clinical groups (MISC_A, MISC_C, COVID-19 and control group) for combination of the six intracellular biomarkers and CD137 for CD4, CD8, CD4$^+$CD8$^+$ and CD4$^-$CD8$^-$. Statistically significant differences (*P*-value ⩽ 0.05) are marked in bold.

		**MISC_A**	**MISC_C**	**COVID-19**	**Control**	***P*-value**					
**CD3$^+$CD4$^+$**	**CD3$^+$CD4$^+$CD137$^+$ /million CD3$^+$**	**24.6 (7.6-523.8)**	**6.4 (0.0-68.0)** [e]	**0.0 (0.0-357.0)**	**492.7 (358.6-593.1)** [e]	0.24	0.24	0.17	0.46	**0.03**[e]	0.06
	**CD3$^+$CD4$^+$GRB$^+$/million CD3$^+$**	**1990.8 (1383.9-4411.5)** [c]	**856.0 (340.8-1345.7)** [d]	**2787.2 (2446.8-5833.3)** [d, f]	**76.4 (0.0-561.5)** [c, f]	0.08	0.15	**0.05**[c]	**0.02**[d]	0.17	**0.02**[f]
	**CD3$^+$CD4$^+$IFNγ$^+$/million CD3$^+$**	97.6 (41.0-292.0)	56.2 (41.3-87.7)	41.5 (5.4-186.2)	30.0 (0.0-72.9)	0.25	0.28	0.12	0.40	0.25	0.28
	**CD3$^+$CD4$^+$IL17$^+$/million CD3$^+$**	**293.0 (256.4-870.9)** [a,b,c]	**50.7 (8.4-140.5)**[a]	**96.7 (89.2-135.4)** [b]	**8.7 (0.0-82.4)** [c]	**0.03**[a]	**0.03**[b]	**0.03**[c]	0.15	0.34	0.07

	**$CD3^+CD4^+IL2^+$/million $CD3^+$**	**217.0 (178.3-291.9)** [b]	167.4 (84.2-193.2)	**33.5 (6.5-102.8)** [b]	40.2 (14.8-220.1)	0.17	**0.03**[b]	0.17	0.15	0.34	0.40
	**$CD3^+CD4^+IL4^+$/million $CD3^+$**	18.9 (7.6-44.6)	0.0 (0.0-0.0)	0.0 (0.0-24.2)	10.7 (2.6-32.9)	0.08	0.24	0.39	0.28	0.13	0.24
	**$CD3^+CD4^+TNF\text{-}\alpha^+$/million $CD3^+$**	145.0 (55.8-219.7)	140.4 (78.4-185.2)	75.8 (17.0-198.7)	53.5 (3.2-111.9)	0.46	0.40	0.21	0.28	0.12	0.40
**$CD3^+CD8^+$**	**$CD3^+CD8^+CD137^+$/million $CD3^+$**	**71.6 (15.3-473-6)** [a]	**0.0 (0.0-0.0)** [a, e]	0.0 (0.0-159.1)	**96.5 (15.0-253.7)** [e]	**0.05**[a]	0.24	0.50	0.12	**0.05**[e]	0.19
	**$CD3^+CD8^+GRB^+$/million $CD3^+$**	8908.0 (5559.3-26271.0)	4752.3 (3719.7-17558.8)	25007.1 (14183.1-35634.3)	5836.4 (0.0-44256.7)	0.17	0.23	0.44	0.12	0.44	0.19
	**$CD3^+CD8^+IFN\gamma^+$/million $CD3^+$**	**162.2 (91.6-273.4)** [b]	30.3 (0.0-92.8)	**41.5 (0.0-77.4)** [b]	36.6 (11.2-97.3)	0.08	**0.03**[b]	0.08	0.50	0.44	0.50
	**$CD3^+CD8^+IL17^+$/million $CD3^+$**	**335.2 (225.8-429.9)** [a, b, c]	**78.0 (31.9-128.9)** [a]	**84.1 (0.0-204.6)**[b]	**33.2 (0.0-114.6)** [c]	**0.05**[a]	**0.05**[b]	**0.05** [c]	0.43	0.34	0.28
	**$CD3^+CD8^+IL2^+$/million $CD3^+$**	**99.2 (62.1-103.6)** [c]	61.0 (28.6-73.9)	35.9 (19.9-61.5)	**12.4 (4.4-23.0)** [c]	0.12	0.12	**0.05**[c]	0.28	0.12	0.08

	**CD3⁺CD8⁺IL4⁺/million CD3⁺**	8.9 (0.0-23.8)	4.1 (1.0-32.9)	0.5 (0.0-23.1)	7.2 (0.0-22.4)	0.44	0.50	0.50	0.28	0.44	0.50
	**CD3⁺CD8⁺TNF-α⁺/million CD3⁺**	50.7 (19.9-113.6)	65.7 (62.2-74.4)	20.7 (5.4-63.3)	0.0 (0.0-61.2)	0.25	0.34	0.19	0.09	0.17	0.18
**CD3⁺CD4⁻CD8⁻**	**CD3⁺CD4⁻CD8⁻CD137⁺/million CD3⁺**	**145.0 (51.4-274.0)** [a]	**0.0 (0.0-0.0)** [a, e]	**0.0 (0.0-33.5)** [f]	**310.4 (180.5-492.9)** [e, f]	**0.05**[a]	0.12	0.12	0.12	**0.03**[e]	**0.03**[f]
	**CD3⁺CD4⁻CD8⁻GRB⁺/million CD3⁺**	13742.7 (9692.3-20500.2)	5531.9 (3473.8-29238.1)	20176.3 (11304.6-31790.1)	12890.8 (7382.9-29996.6)	0.17	0.19	0.50	0.09	0.34	0.28
	**CD3⁺CD4⁻CD8⁻IFNγ⁺/million CD3⁺**	**168.4 (104.7-233.8)** [c]	32.7 (14.4-133.2)	56.7 (10.5-219.5)	**12.3 (0.2-35.2)** [c]	0.12	0.23	**0.03**[c]	0.47	0.29	0.23
	**CD3⁺CD4⁻CD8⁻IL17⁺/million CD3⁺**	99.8 (36.3-238.2)	54.7 (16.6-133.6)	35.5 (0.0-205.9)	111.4 (37.0-186.7)	0.39	0.37	0.50	0.50	0.39	0.43
	**CD3⁺CD4⁻CD8⁻IL2⁺/million CD3⁺**	**146.9 (84.3-164.5)** [a]	**25.3 (9.5-34.0)** [a]	35.7 (14.6-204.0)	50.8 (0.0-103.1)	**0.03**[a]	0.19	0.08	0.21	0.44	0.34
	**CD3⁺CD4⁻CD8⁻IL4⁺/million CD3⁺**	12.6 (0.0-54.3)	**32.3 (10.9-55.9)** [d]	**0.0 (0.0-17.1)** [d]	16.1 (0.0-36.9)	0.34	0.21	0.50	**0.05**[d]	0.17	0.21

	**$CD3^+CD4^-CD8^-TNF\text{-}\alpha^+$/million $CD3^+$**	69.3 (47.8-148.1)	29.4 (5.7-56.5)	8.3 (0.0-139.6)	0.4 (0.0-125.7)	0.12	0.11	0.17	0.43	0.34	0.28
	**$CD3^+CD4^+CD8^+CD137^+$/million $CD3^+$**	3.5 (0.2-24.4)	**0.0 (0.0-3.6)** [e]	2.5 (0.0-21.0)	**30.0 (24.8-33.3)** [e]	0.19	0.37	0.17	0.19	**0.03**[e]	0.11
	**$CD3^+CD4^+CD8^+GRB^+$/million $CD3^+$**	29.7 (10.8-311.3)	138.8 (67.8-168.2)	1.4 (0.0-93.4)	75.1 (26.3-115.9)	0.39	0.33	0.34	0.14	0.17	0.18
	**$CD3^+CD4^+CD8^+IL17^+$/million $CD3^+$**	**0.0 (0.0-0.0)** [c]	0.0 (0.0-14.1)	0.2 (0.0-38.6)	**20.2 (10.1-29.4)** [c]	0.24	0.08	**0.03**[c]	0.26	0.07	0.11
**$CD3^+CD4^+CD8^+$**	**$CD3^+CD4^+CD8^+IFN\gamma^+$/million $CD3^+$**	**0.0 (0.0-0.0)** [b,c]	5.1 (0.0-18.2)	**3.8 (1.3-27.1)** [b]	**23.6 (7.1-70.1)** [c]	0.11	**0.02**[b]	**0.03**[c]	0.28	0.12	0.15
	**$CD3^+CD4^+CD8^+IL2^+$/million $CD3^+$**	5.8 (1.7-26.8)	9.2 (7.6-11.4)	13.3 (2.4-24.5)	20.3 (8.2-48.7)	0.25	0.34	0.17	0.34	0.34	0.28
	**$CD3^+CD4^+CD8^+IL4^+$/million $CD3^+$**	**11.7 (5.8-17.7)** [b]	3.8 (0.0-8.0)	**0.0 (0.0-6.0)** [b]	2.4 (0.0-11.7)	0.11	**0.05**[b]	0.24	0.46	0.50	0.32
	**$CD3^+CD4^+CD8^+TNF\text{-}\alpha^+$/million $CD3^+$**	22.9 (7.6-40.6)	8.2 (5.1-17.5)	2.4 (1.0-12.1)	1.5 (0.4-16.6)	0.17	0.07	0.08	0.15	0.17	0.40

Abbreviations: MISC_A: Multisystem Inflammatory Syndrome in Children (MIS-C) in acute phase of the syndrome; MISC_C: MIS-C in the convalescent phase of the syndrome; GRB: Granzyme B; IL17: Interleukin 17; IFNγ: Interferon-gamma; IL2: Interleukin 2; IL4: Interleukin 4; TNF-α: Tumor Necrosis Factor- α.

[a]: MISC_A vs MISC_C,[b]: MISC_A vs COVID-19,[c]: MISC_A vs Control,[d]: MISC_C vs COVID-19,[e]: MISC_C vs Control,[f]: COVID-19 vs Control.

Supplementary Figure S1. Flow cytometry gating strategy for SARS-CoV-2 Spike-protein specific PBMCs expression of $CD4^{+}$IL-17 in stimulated (left) and unstimulated (right) samples of each MISC_A (Panel A), MISC_C (Panel B) and COVID-19 (Panel C). Blue colors represent $CD4^{+}$ T-cells and pink colors represent $CD4^{+}CD137^{+}$ T-cells.

Panel A.

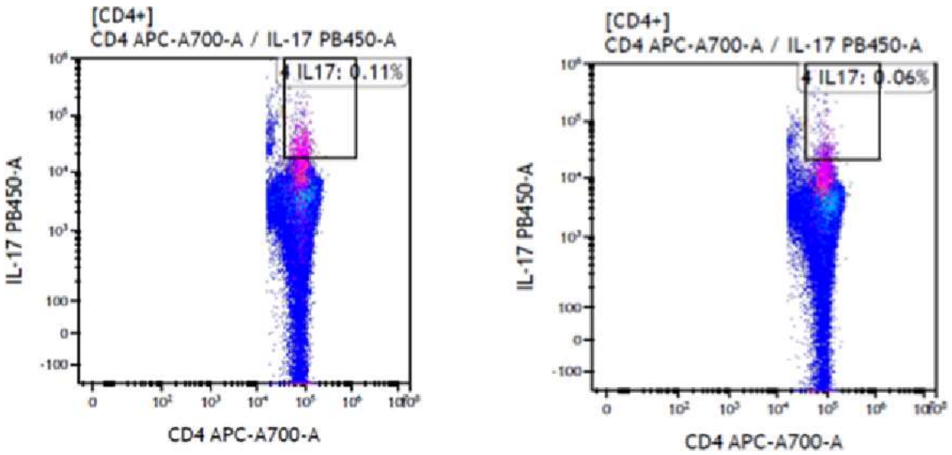

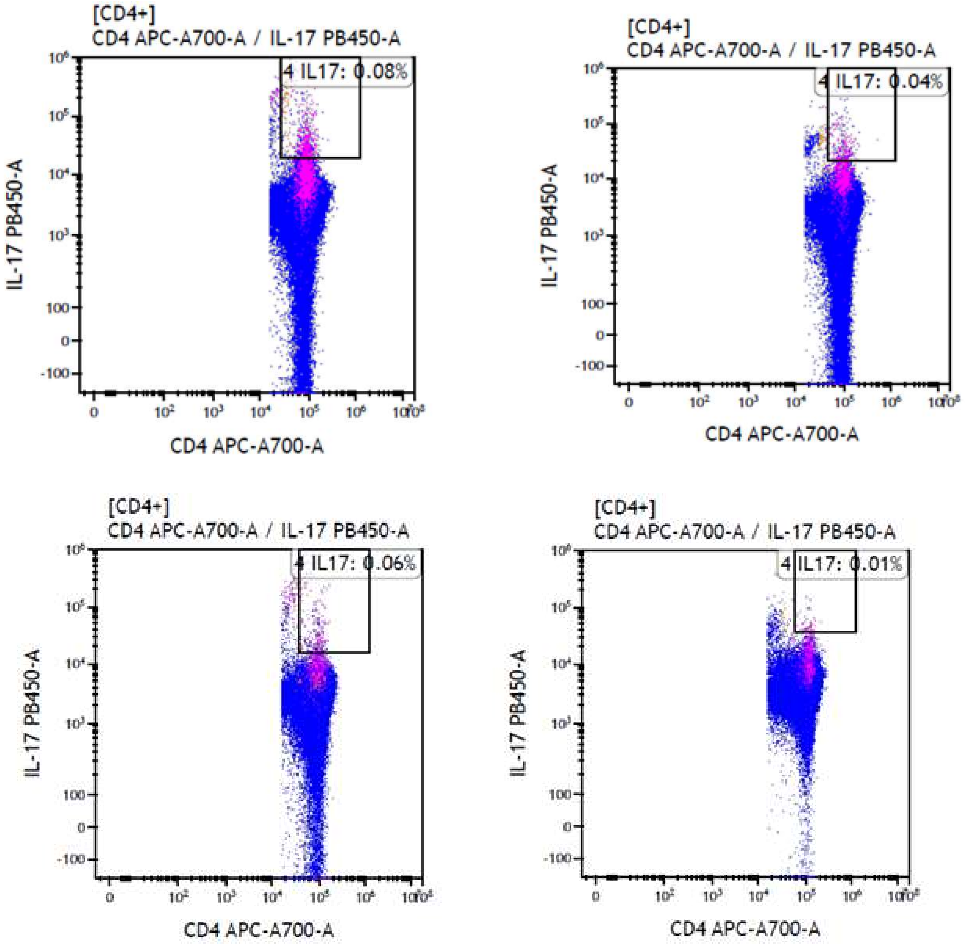
[CD4+]
CD4 APC-A700-A / IL-17 PB450-A
4 IL17: 0.08%
IL-17 PB450-A
CD4 APC-A700-A
[CD4+]
CD4 APC-A700-A / IL-17 PB450-A
4 IL17: 0.04%
IL-17 PB450-A
CD4 APC-A700-A
[CD4+]
CD4 APC-A700-A / IL-17 PB450-A
4 IL17: 0.06%
IL-17 PB450-A
CD4 APC-A700-A
[CD4+]
CD4 APC-A700-A / IL-17 PB450-A
4 IL17: 0.01%
IL-17 PB450-A
CD4 APC-A700-A

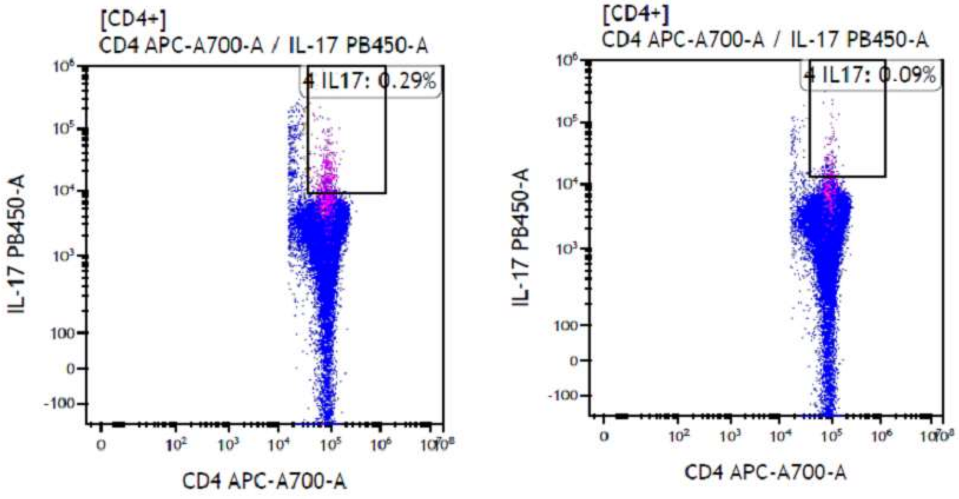
[CD4+]
CD4 APC-A700-A / IL-17 PB450-A
4 IL17: 0.29%
IL-17 PB450-A
CD4 APC-A700-A
[CD4+]
CD4 APC-A700-A / IL-17 PB450-A
4 IL17: 0.09%
IL-17 PB450-A
CD4 APC-A700-A

Panel B.

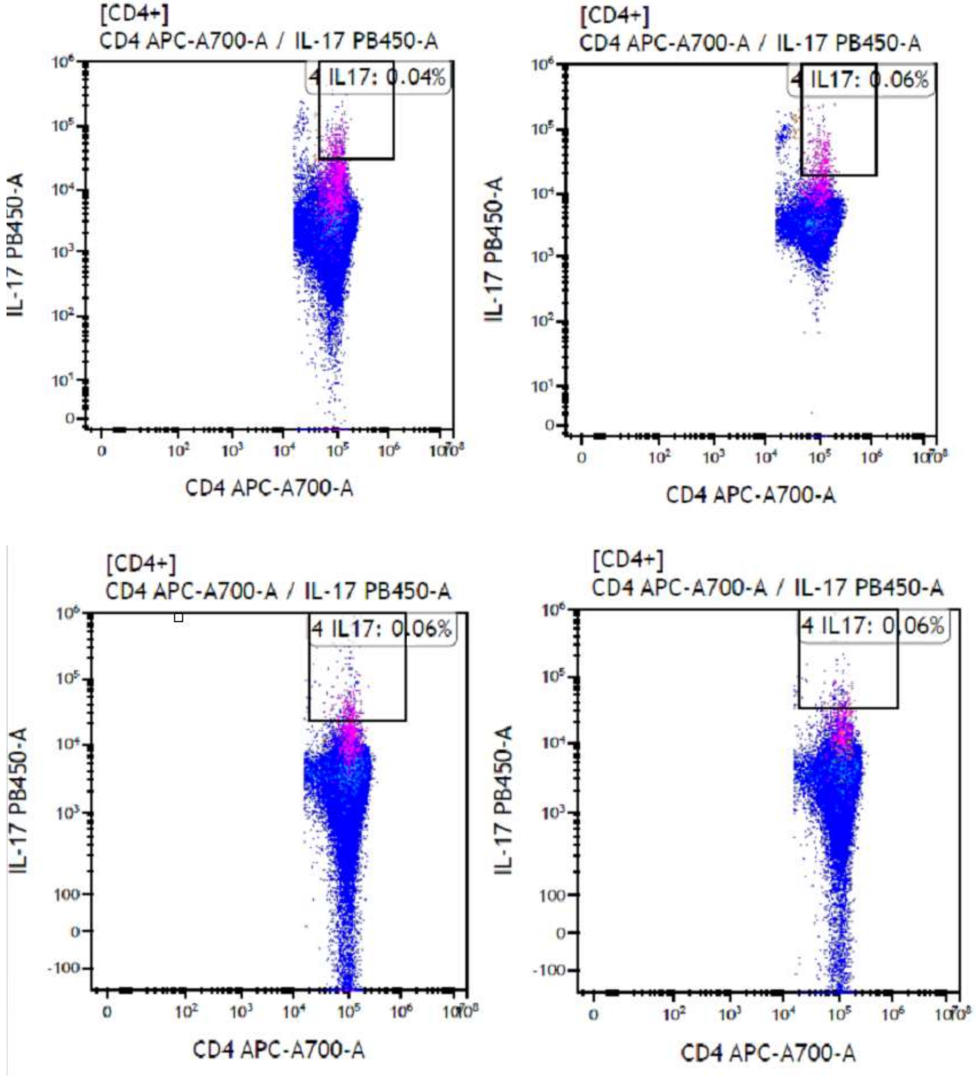
[CD4+]
CD4 APC-A700-A / IL-17 PB450-A
IL17: 0.04%
IL-17 PB450-A
CD4 APC-A700-A
[CD4+]
CD4 APC-A700-A / IL-17 PB450-A
IL17: 0.06%
IL-17 PB450-A
CD4 APC-A700-A
[CD4+]
CD4 APC-A700-A / IL-17 PB450-A
IL17: 0.06%
IL-17 PB450-A
CD4 APC-A700-A
[CD4+]
CD4 APC-A700-A / IL-17 PB450-A
IL17: 0.06%
IL-17 PB450-A
CD4 APC-A700-A

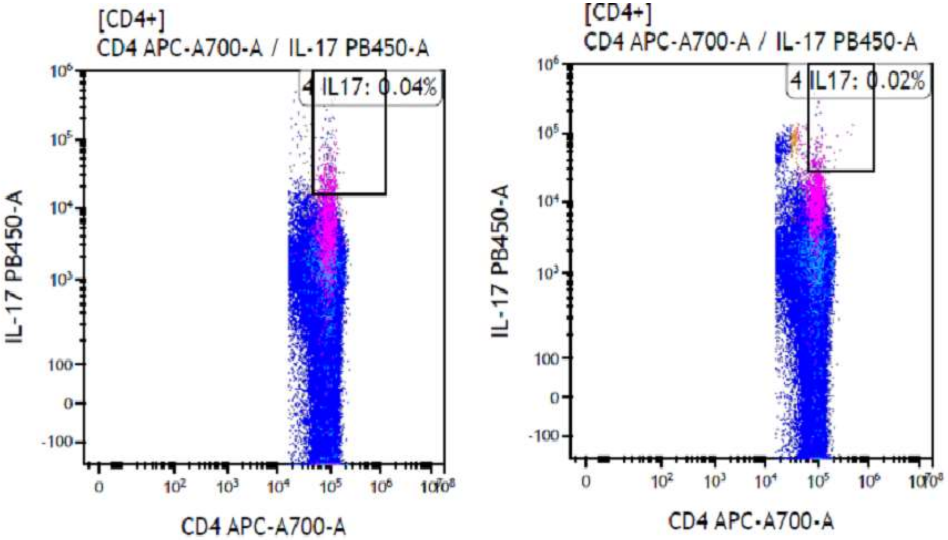
[CD4+]
CD4 APC-A700-A / IL-17 PB450-A
4 IL17: 0.04%
IL-17 PB450-A
CD4 APC-A700-A
[CD4+]
CD4 APC-A700-A / IL-17 PB450-A
4 IL17: 0.02%
IL-17 PB450-A
CD4 APC-A700-A

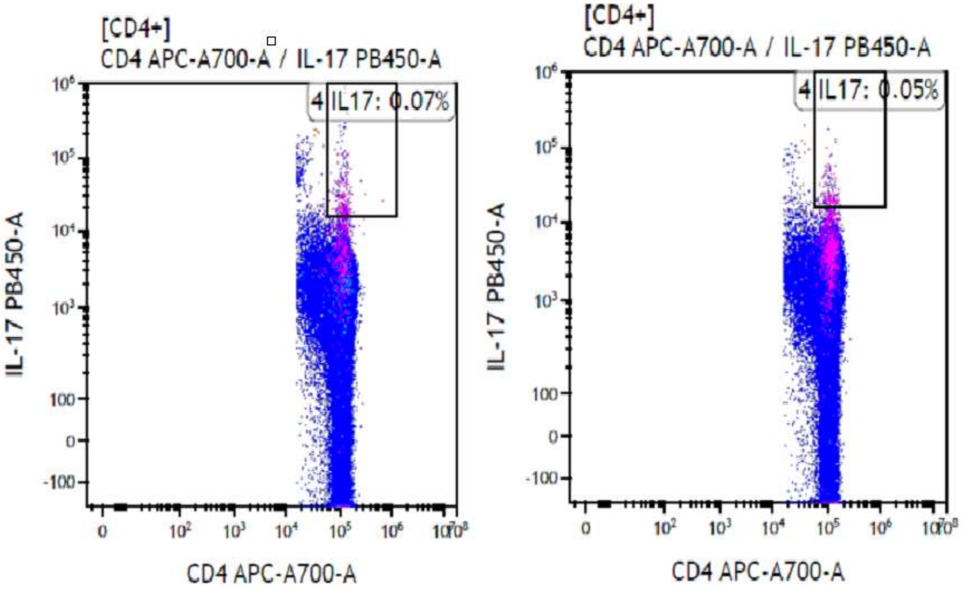
[CD4+]
CD4 APC-A700-A / IL-17 PB450-A
4 IL17: 0.07%
IL-17 PB450-A
CD4 APC-A700-A
[CD4+]
CD4 APC-A700-A / IL-17 PB450-A
4 IL17: 0.05%
IL-17 PB450-A
CD4 APC-A700-A

Panel C.

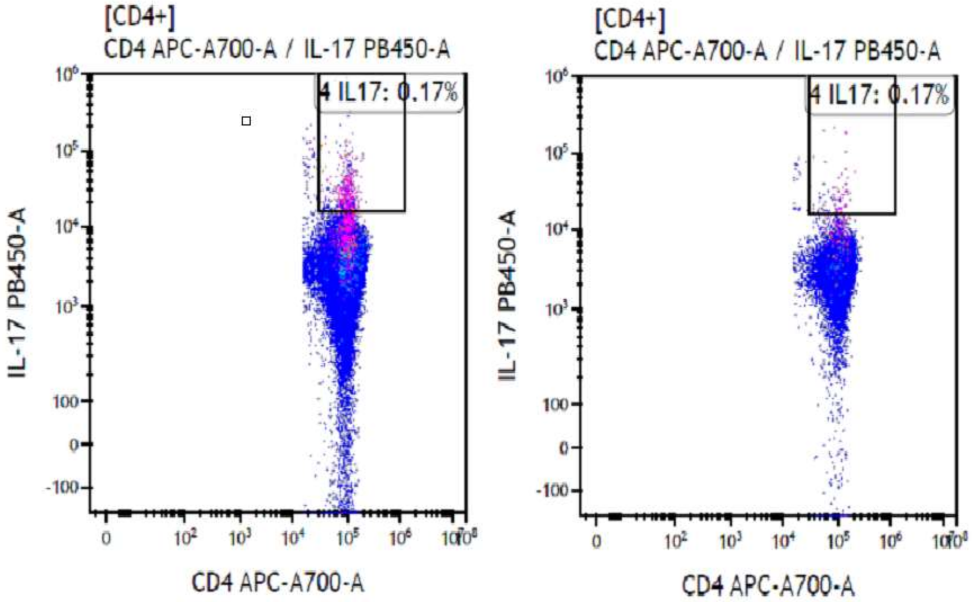
[CD4+]
CD4 APC-A700-A / IL-17 PB450-A
4 IL17: 0.17%
IL-17 PB450-A
CD4 APC-A700-A
[CD4+]
CD4 APC-A700-A / IL-17 PB450-A
4 IL17: 0.17%
IL-17 PB450-A
CD4 APC-A700-A

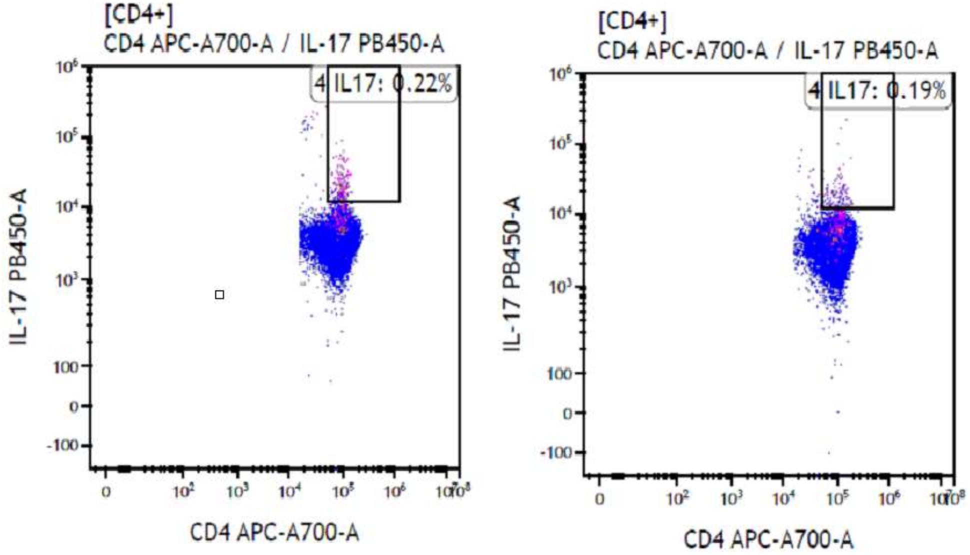
[CD4+]
CD4 APC-A700-A / IL-17 PB450-A
4 IL17: 0.22%
IL-17 PB450-A
CD4 APC-A700-A
[CD4+]
CD4 APC-A700-A / IL-17 PB450-A
4 IL17: 0.19%
IL-17 PB450-A
CD4 APC-A700-A

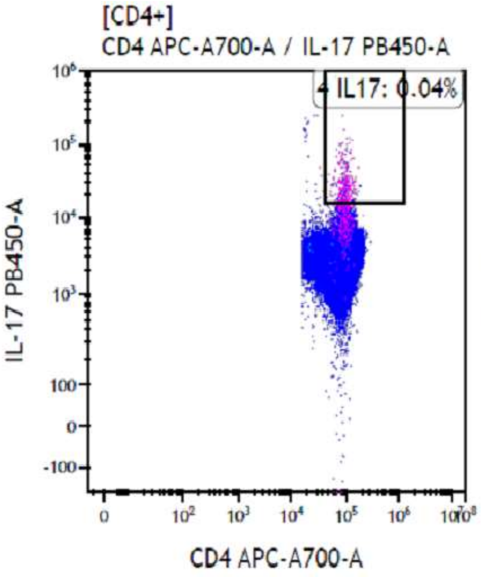
[CD4+]
CD4 APC-A700-A / IL-17 PB450-A
4 IL17: 0.04%
IL-17 PB450-A
CD4 APC-A700-A

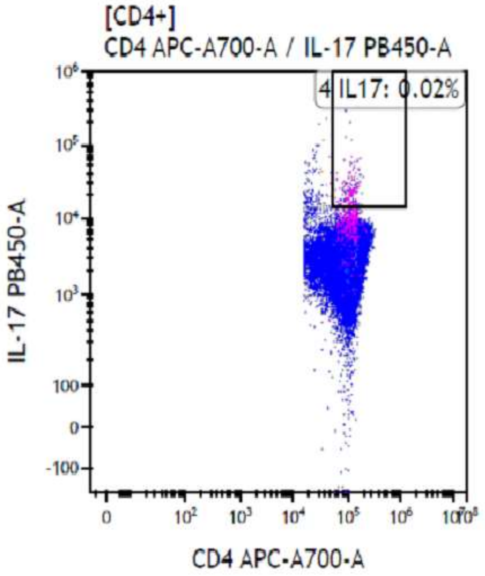
[CD4+]
CD4 APC-A700-A / IL-17 PB450-A
4 IL17: 0.02%
IL-17 PB450-A
CD4 APC-A700-A

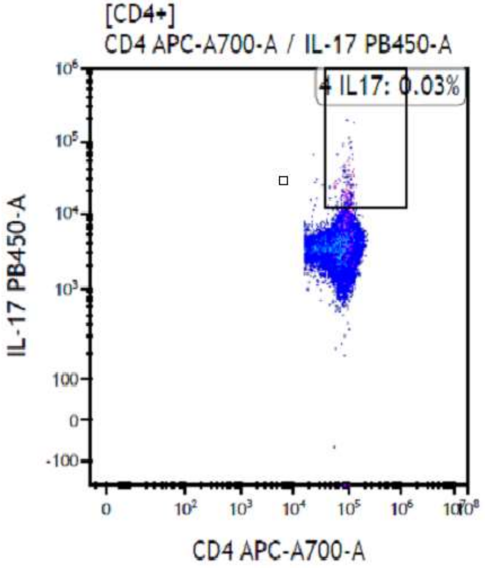
[CD4+]
CD4 APC-A700-A / IL-17 PB450-A
4 IL17: 0.03%
IL-17 PB450-A
CD4 APC-A700-A

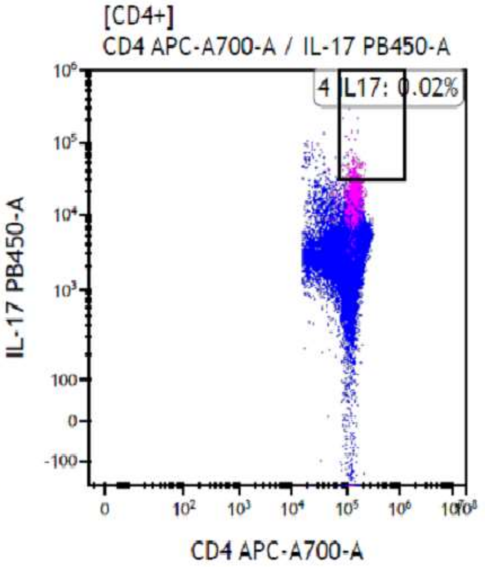
[CD4+]
CD4 APC-A700-A / IL-17 PB450-A
4 IL17: 0.02%
IL-17 PB450-A
CD4 APC-A700-A

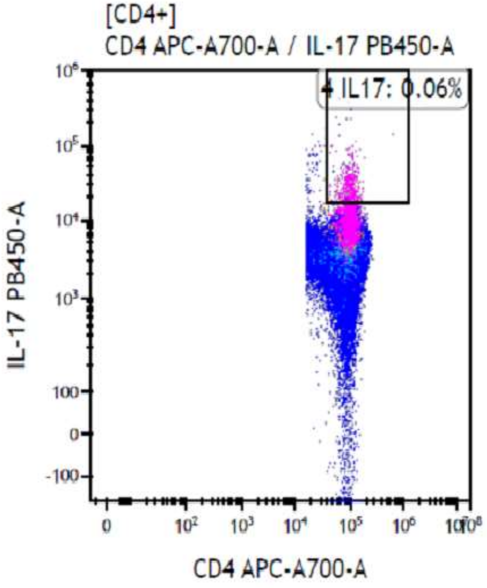
[CD4+]
CD4 APC-A700-A / IL-17 PB450-A
4 IL17: 0.06%
IL-17 PB450-A
CD4 APC-A700-A

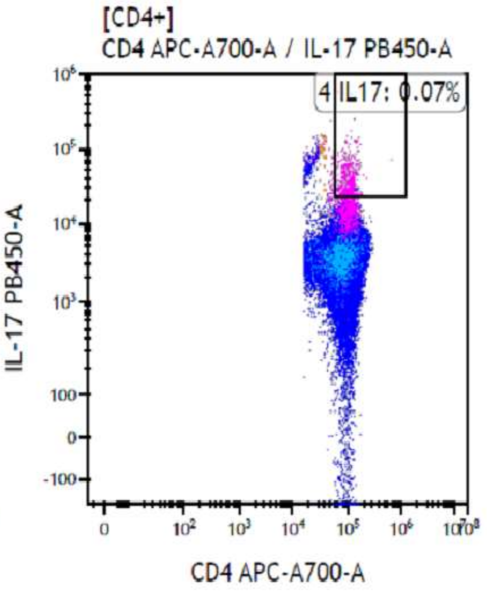
[CD4+]
CD4 APC-A700-A / IL-17 PB450-A
4 IL17: 0.07%
IL-17 PB450-A
CD4 APC-A700-A

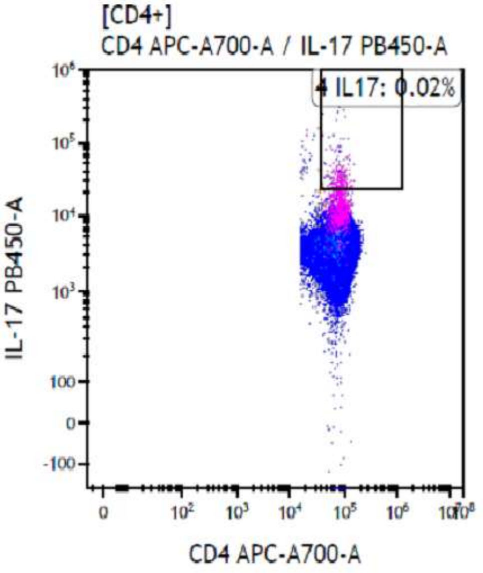
[CD4+]
CD4 APC-A700-A / IL-17 PB450-A
IL17: 0.02%
IL-17 PB450-A
CD4 APC-A700-A

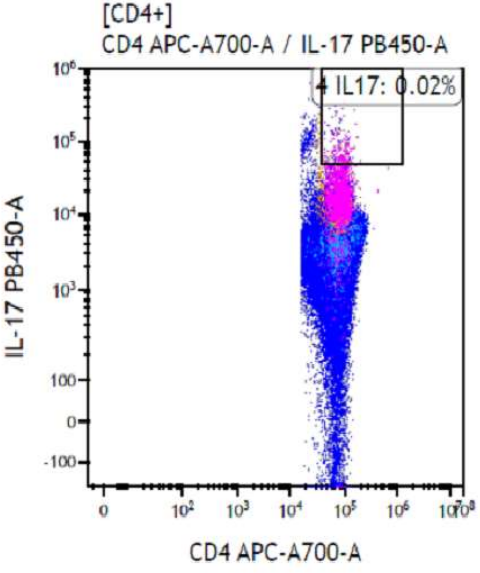
[CD4+]
CD4 APC-A700-A / IL-17 PB450-A
IL17: 0.02%
IL-17 PB450-A
CD4 APC-A700-A

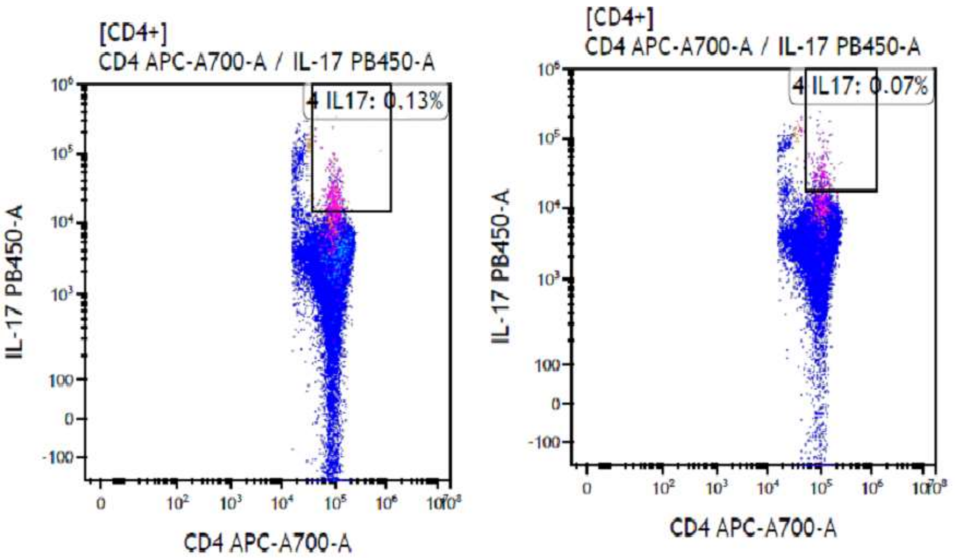
[CD4+]
CD4 APC-A700-A / IL-17 PB450-A
4 IL17: 0.13%
IL-17 PB450-A
CD4 APC-A700-A
[CD4+]
CD4 APC-A700-A / IL-17 PB450-A
4 IL17: 0.07%
IL-17 PB450-A
CD4 APC-A700-A

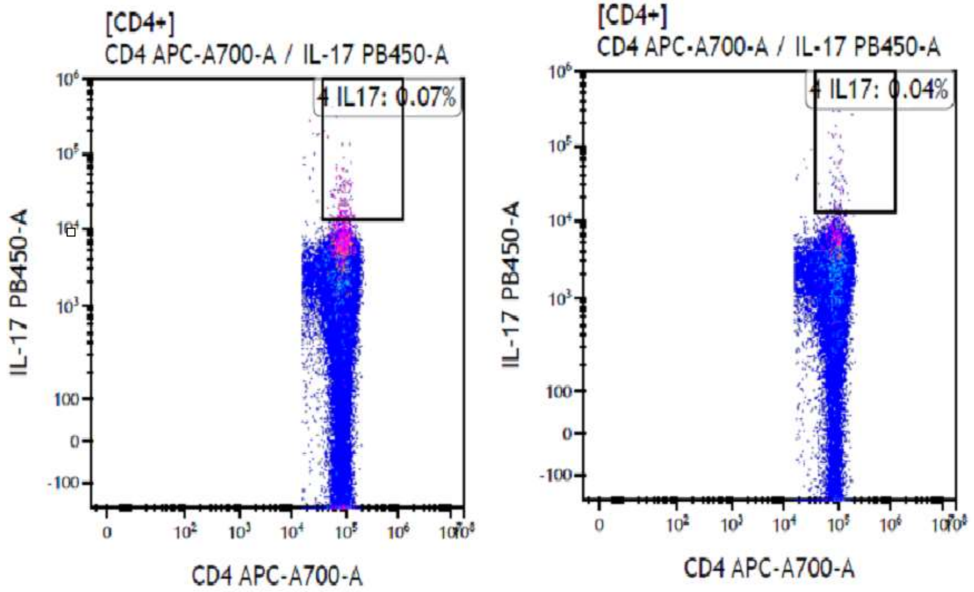
[CD4+]
CD4 APC-A700-A / IL-17 PB450-A
4 IL17: 0.07%
IL-17 PB450-A
CD4 APC-A700-A
[CD4+]
CD4 APC-A700-A / IL-17 PB450-A
4 IL17: 0.04%
IL-17 PB450-A
CD4 APC-A700-A

Panel D.

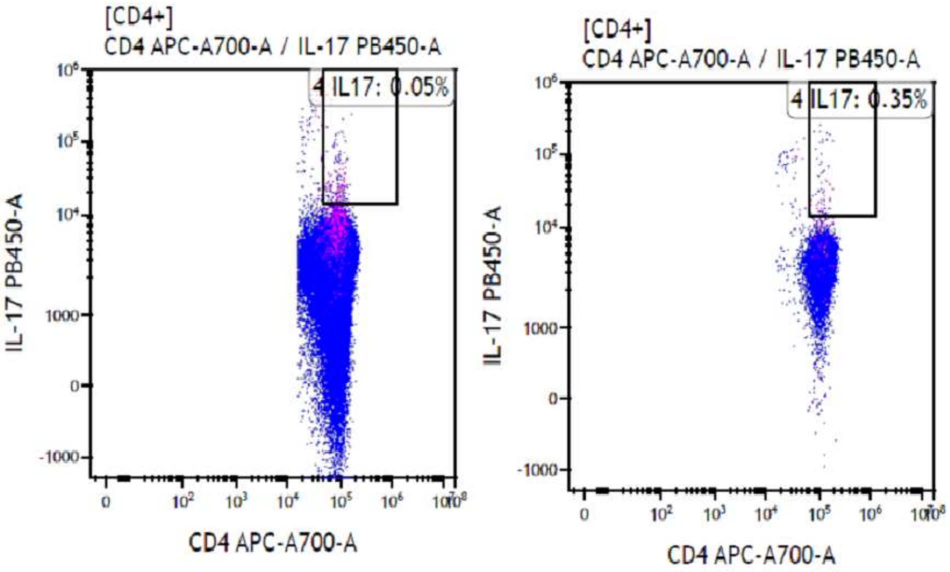
[CD4+]
CD4 APC-A700-A / IL-17 PB450-A
4 IL17: 0.05%
IL-17 PB450-A
CD4 APC-A700-A
[CD4+]
CD4 APC-A700-A / IL-17 PB450-A
4 IL17: 0.35%
IL-17 PB450-A
CD4 APC-A700-A

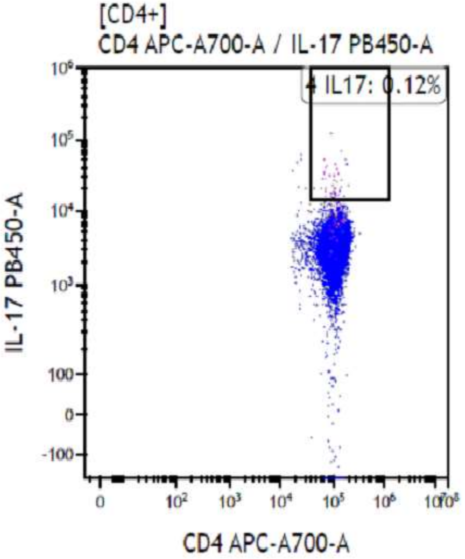
[CD4+]
CD4 APC-A700-A / IL-17 PB450-A
4 IL17: 0.12%
IL-17 PB450-A
CD4 APC-A700-A

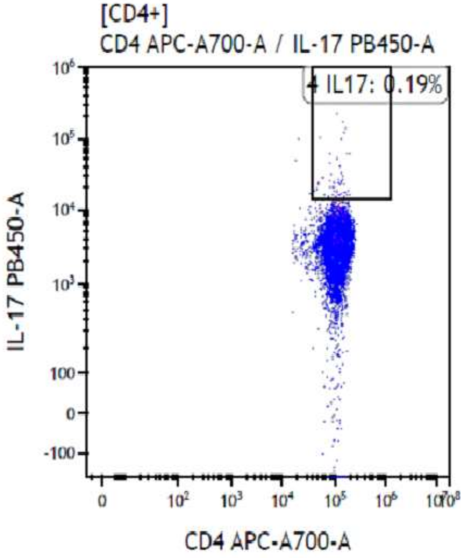
[CD4+]
CD4 APC-A700-A / IL-17 PB450-A
4 IL17: 0.19%
IL-17 PB450-A
CD4 APC-A700-A

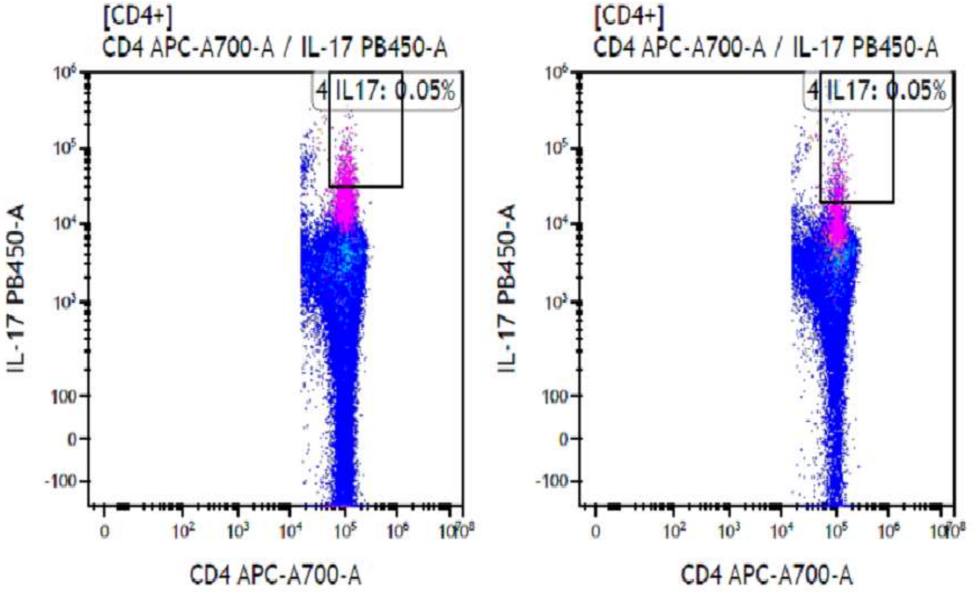
[CD4+]
CD4 APC-A700-A / IL-17 PB450-A
4 IL17: 0.05%
IL-17 PB450-A
CD4 APC-A700-A
[CD4+]
CD4 APC-A700-A / IL-17 PB450-A
4 IL17: 0.05%
IL-17 PB450-A
CD4 APC-A700-A

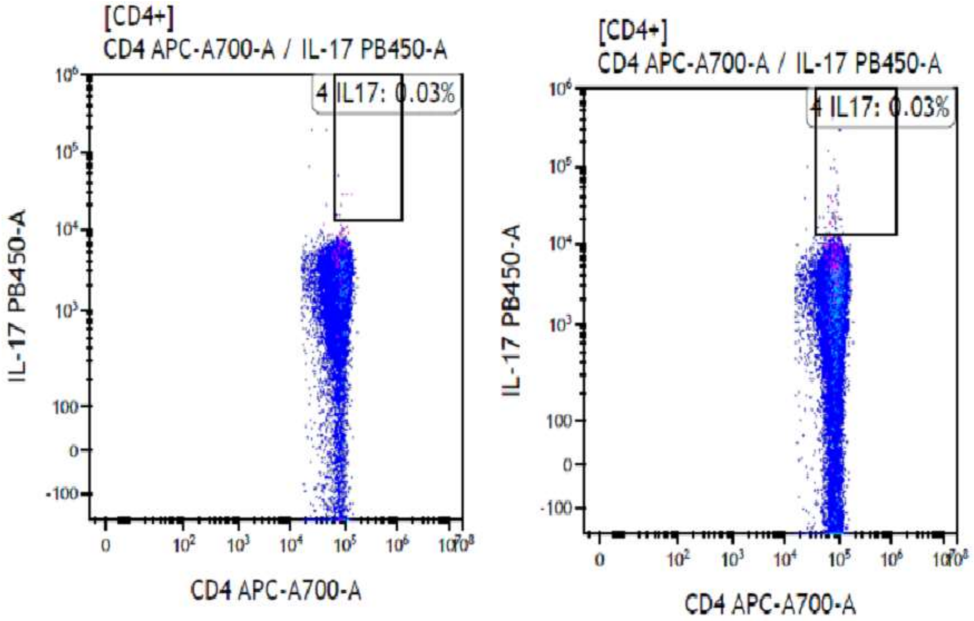

Supplementary Figure S2. Flow cytometry gating strategy for SARS-CoV-2 Spike-protein specific PBMCs expression of CD8[+]IL-17 in stimulated (left) and unstimulated (right) samples of each MISC_A (Panel A), MISC_C (Panel B) and COVID-19 (Panel C).

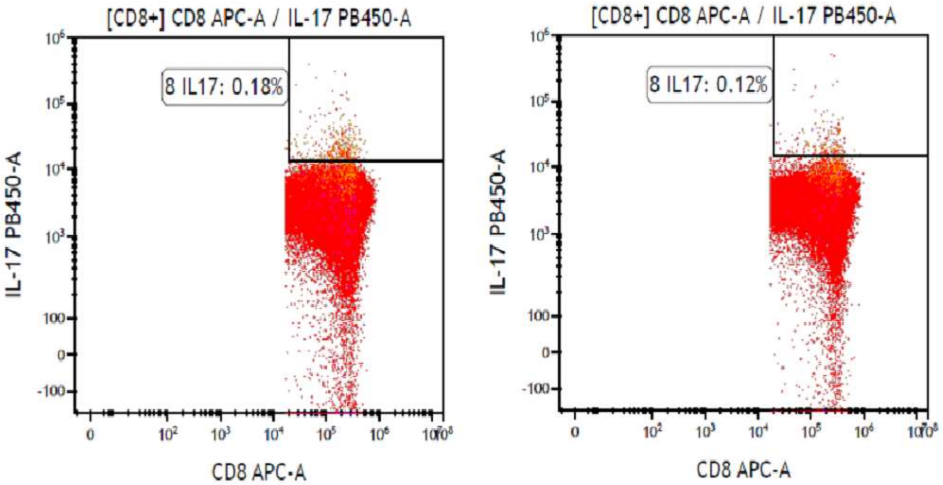
[CD8+] CD8 APC-A / IL-17 PB450-A
8 IL17: 0.18%
IL-17 PB450-A
CD8 APC-A
[CD8+] CD8 APC-A / IL-17 PB450-A
8 IL17: 0.12%
IL-17 PB450-A
CD8 APC-A

Panel A.

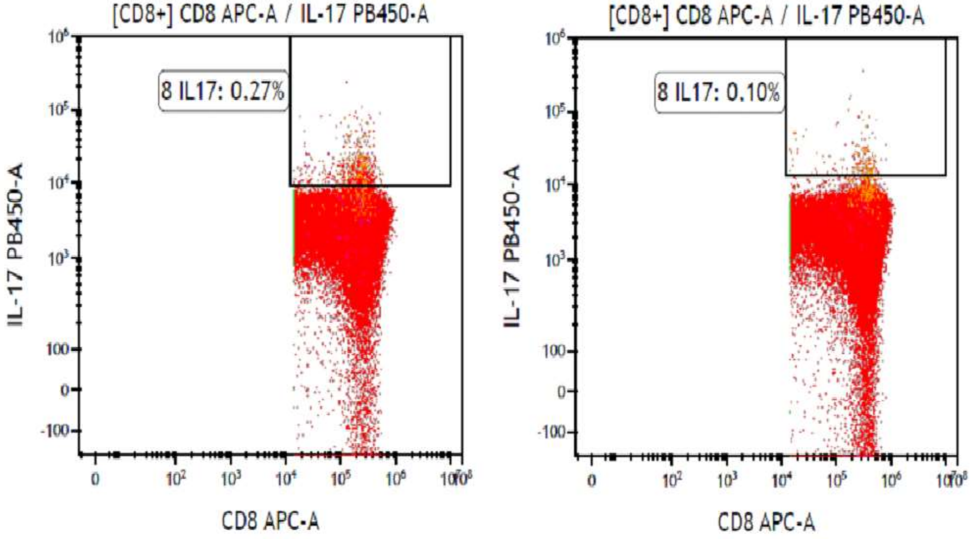
[CD8+] CD8 APC-A / IL-17 PB450-A
8 IL17: 0.27%
IL-17 PB450-A
CD8 APC-A
[CD8+] CD8 APC-A / IL-17 PB450-A
8 IL17: 0.10%
IL-17 PB450-A
CD8 APC-A

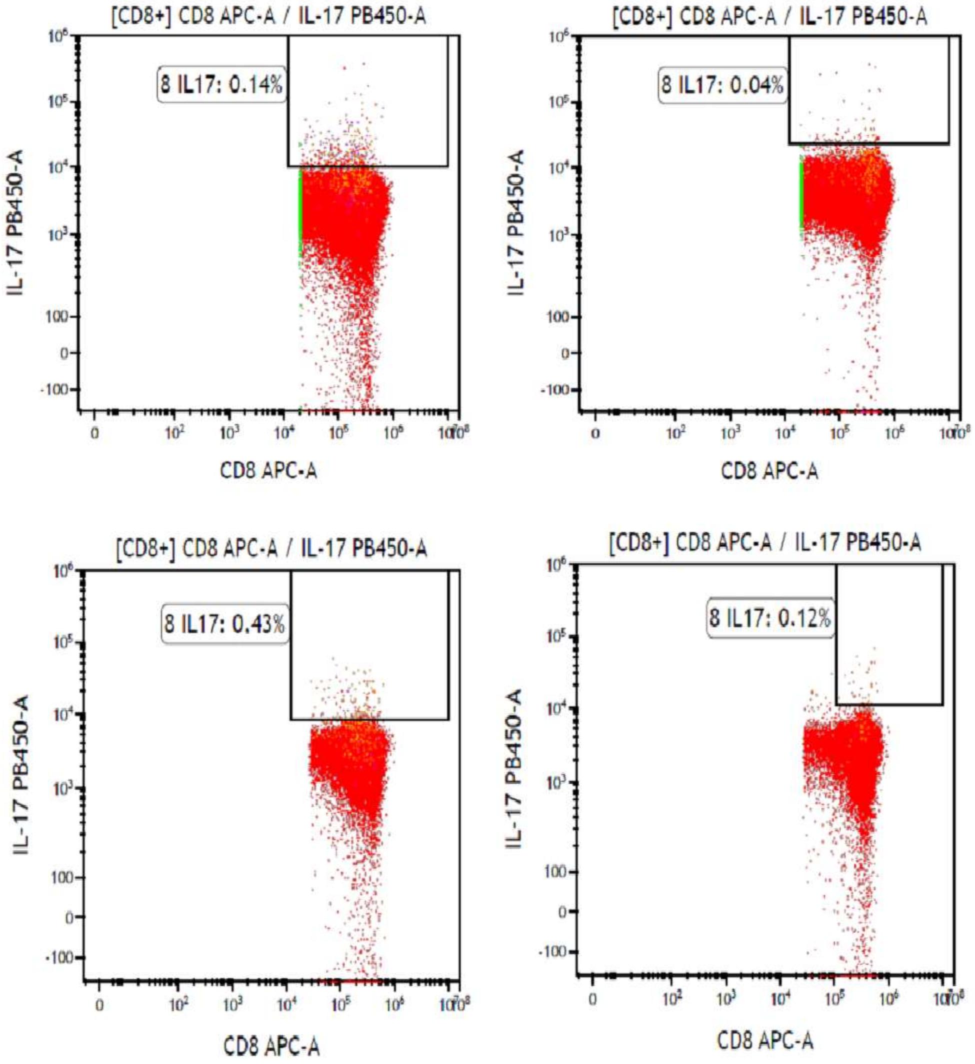
[CD8+] CD8 APC-A / IL-17 PB450-A
8 IL17: 0.14%
IL-17 PB450-A
CD8 APC-A
[CD8+] CD8 APC-A / IL-17 PB450-A
8 IL17: 0.04%
IL-17 PB450-A
CD8 APC-A
[CD8+] CD8 APC-A / IL-17 PB450-A
8 IL17: 0.43%
IL-17 PB450-A
CD8 APC-A
[CD8+] CD8 APC-A / IL-17 PB450-A
8 IL17: 0.12%
IL-17 PB450-A
CD8 APC-A

Panel B.

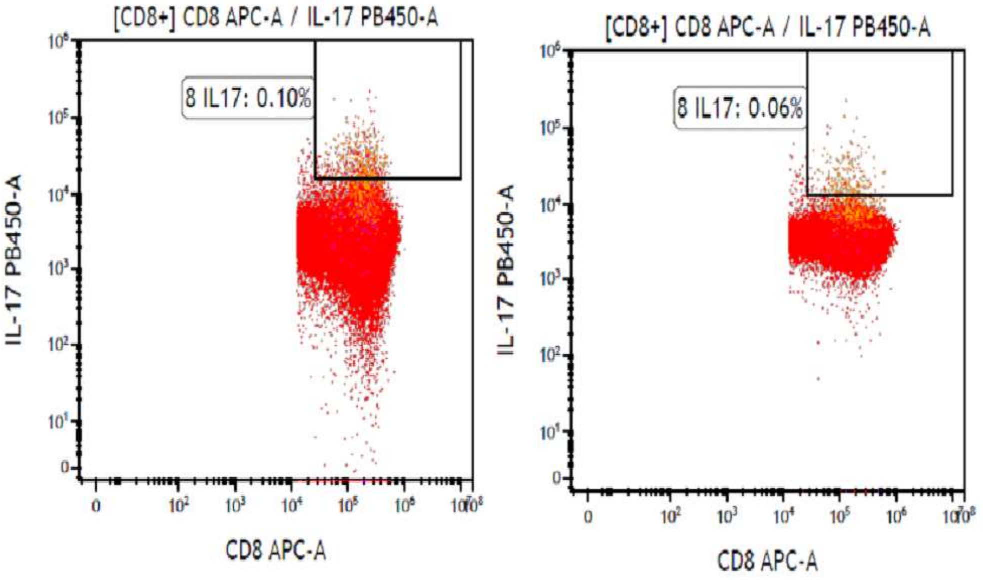
[CD8+] CD8 APC-A / IL-17 PB450-A
8 IL17: 0.10%
IL-17 PB450-A
CD8 APC-A
[CD8+] CD8 APC-A / IL-17 PB450-A
8 IL17: 0.06%
IL-17 PB450-A
CD8 APC-A

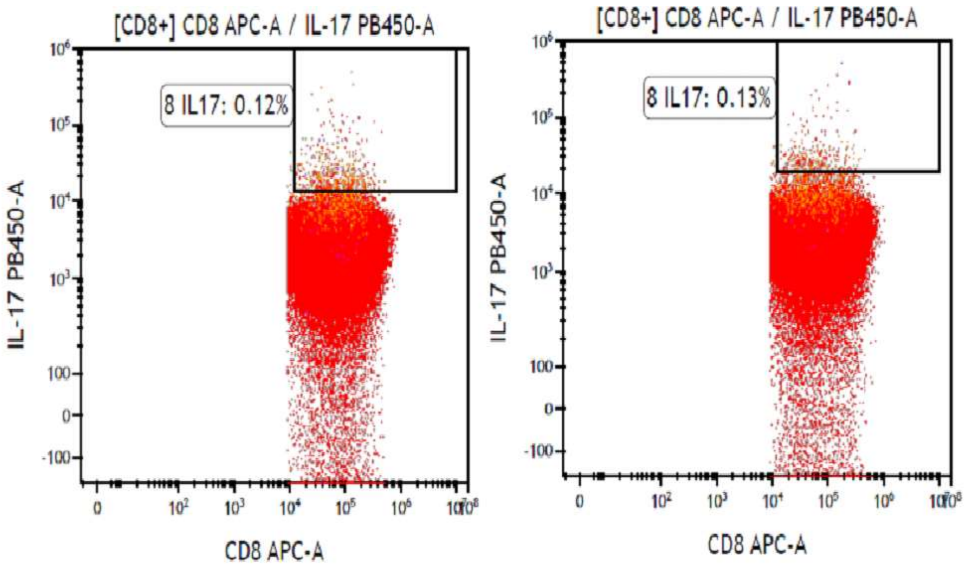
[CD8+] CD8 APC-A / IL-17 PB450-A
8 IL17: 0.12%
IL-17 PB450-A
CD8 APC-A
[CD8+] CD8 APC-A / IL-17 PB450-A
8 IL17: 0.13%
IL-17 PB450-A
CD8 APC-A

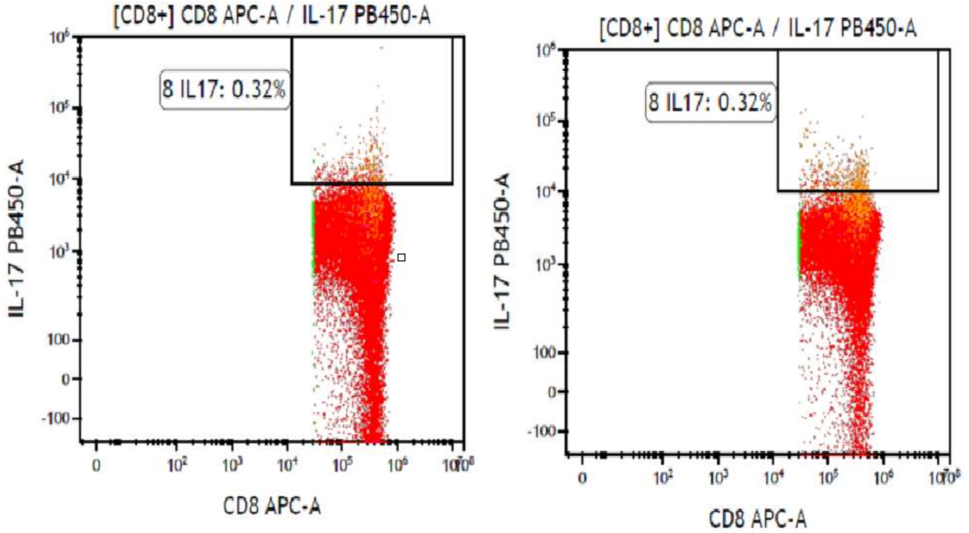
[CD8+] CD8 APC-A / IL-17 PB450-A
8 IL17: 0.32%
IL-17 PB450-A
CD8 APC-A
[CD8+] CD8 APC-A / IL-17 PB450-A
8 IL17: 0.32%
IL-17 PB450-A
CD8 APC-A

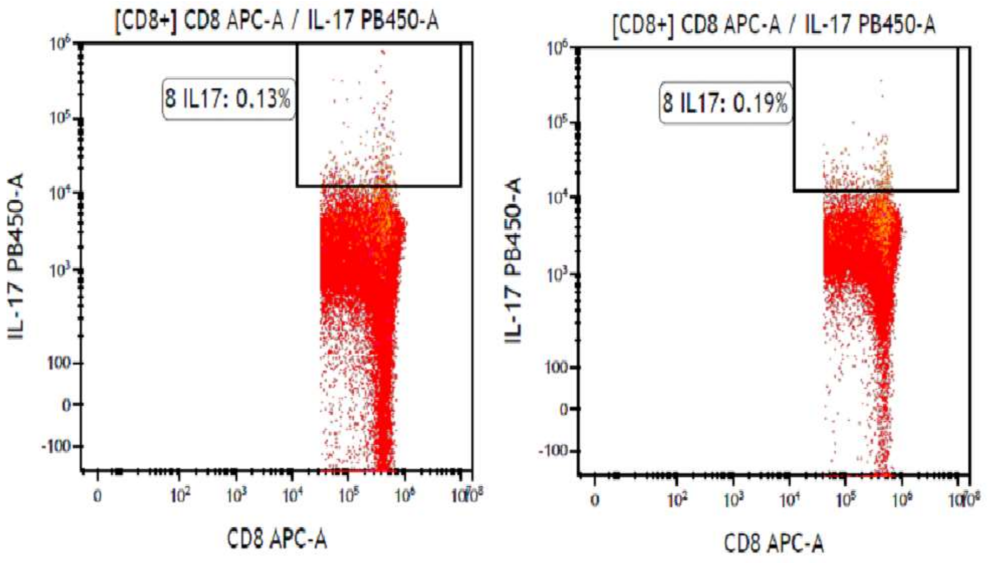
[CD8+] CD8 APC-A / IL-17 PB450-A
8 IL17: 0.13%
IL-17 PB450-A
CD8 APC-A
[CD8+] CD8 APC-A / IL-17 PB450-A
8 IL17: 0.19%
IL-17 PB450-A
CD8 APC-A

Panel C.

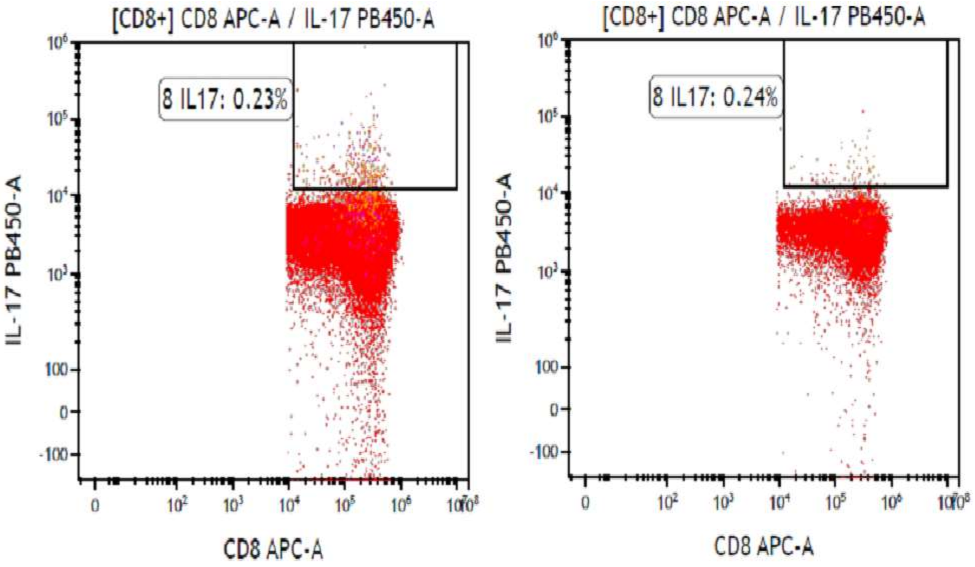
[CD8+] CD8 APC-A / IL-17 PB450-A
8 IL17: 0.23%
IL-17 PB450-A
CD8 APC-A
[CD8+] CD8 APC-A / IL-17 PB450-A
8 IL17: 0.24%
IL-17 PB450-A
CD8 APC-A

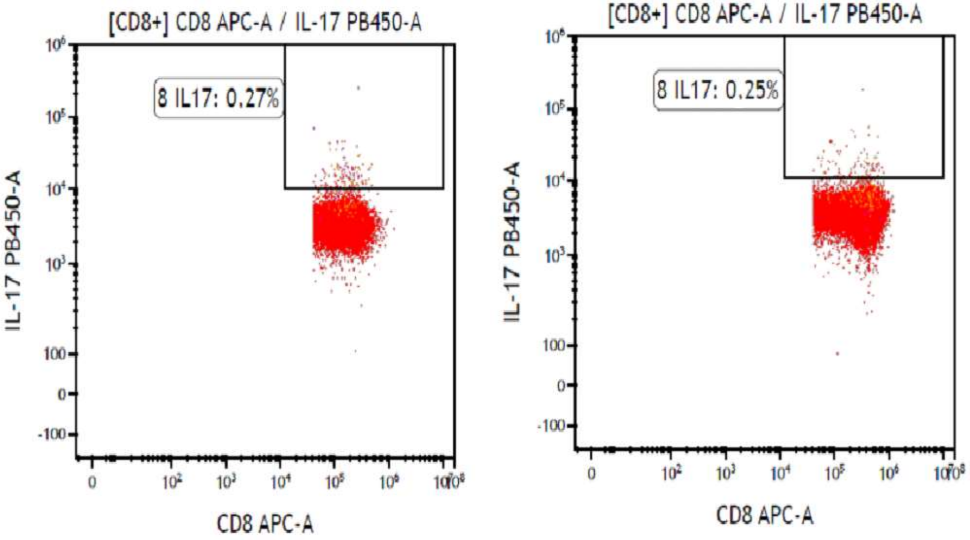
[CD8+] CD8 APC-A / IL-17 PB450-A
8 IL17: 0.27%
IL-17 PB450-A
CD8 APC-A
[CD8+] CD8 APC-A / IL-17 PB450-A
8 IL17: 0.25%
IL-17 PB450-A
CD8 APC-A

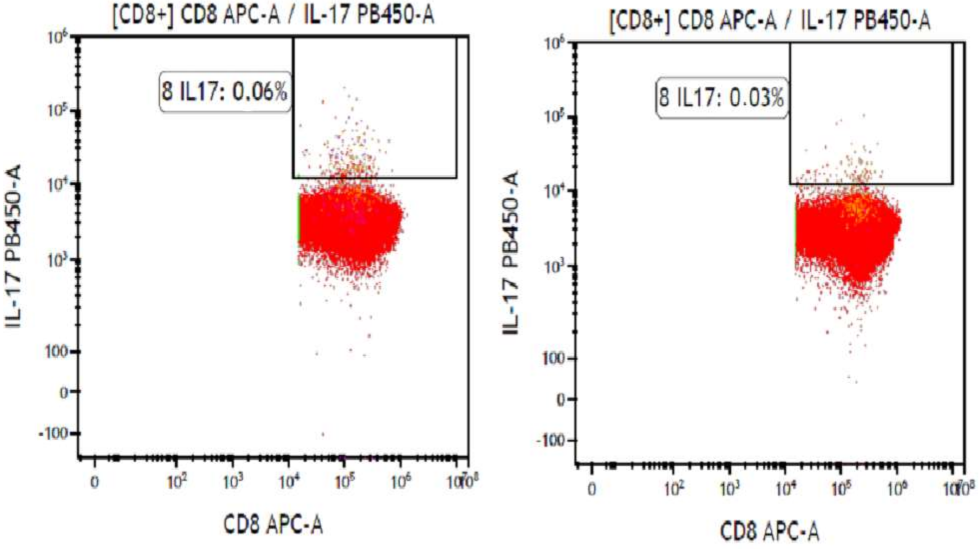
[CD8+] CD8 APC-A / IL-17 PB450-A
8 IL17: 0.06%
IL-17 PB450-A
CD8 APC-A
[CD8+] CD8 APC-A / IL-17 PB450-A
8 IL17: 0.03%
IL-17 PB450-A
CD8 APC-A

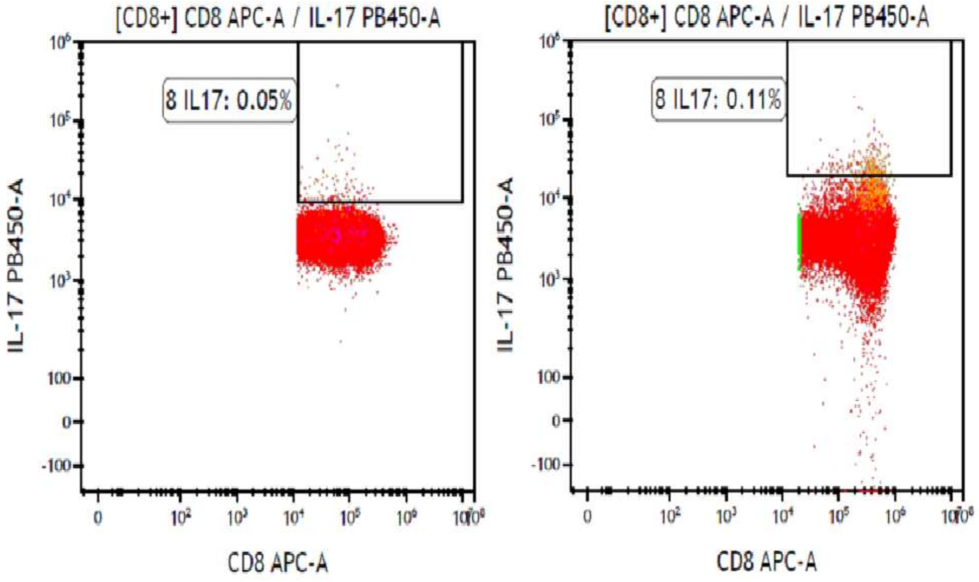
[CD8+] CD8 APC-A / IL-17 PB450-A
8 IL17: 0.05%
IL-17 PB450-A
CD8 APC-A
[CD8+] CD8 APC-A / IL-17 PB450-A
8 IL17: 0.11%
IL-17 PB450-A
CD8 APC-A

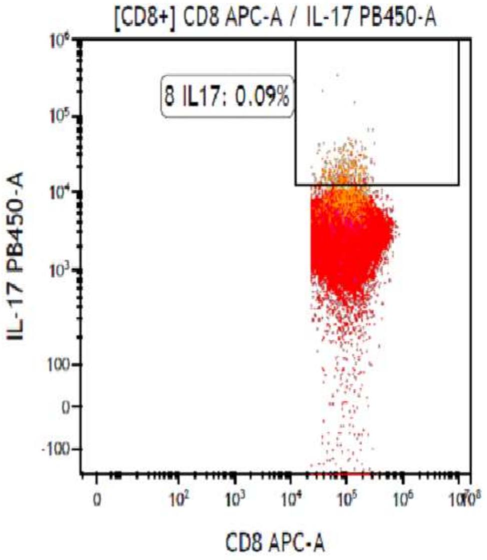
[CD8+] CD8 APC-A / IL-17 PB450-A
8 IL17: 0.09%
IL-17 PB450-A
CD8 APC-A

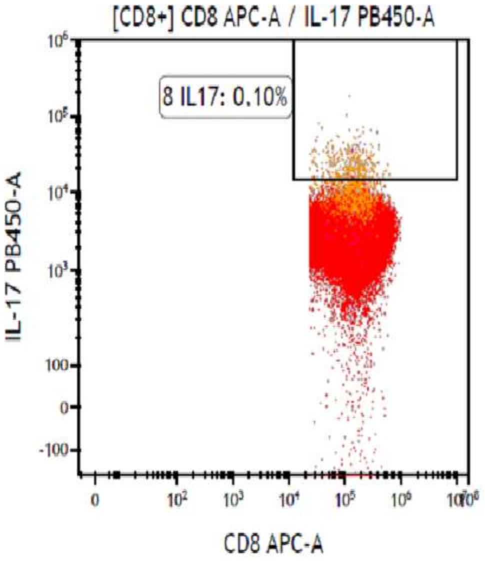
[CD8+] CD8 APC-A / IL-17 PB450-A
8 IL17: 0.10%
IL-17 PB450-A
CD8 APC-A

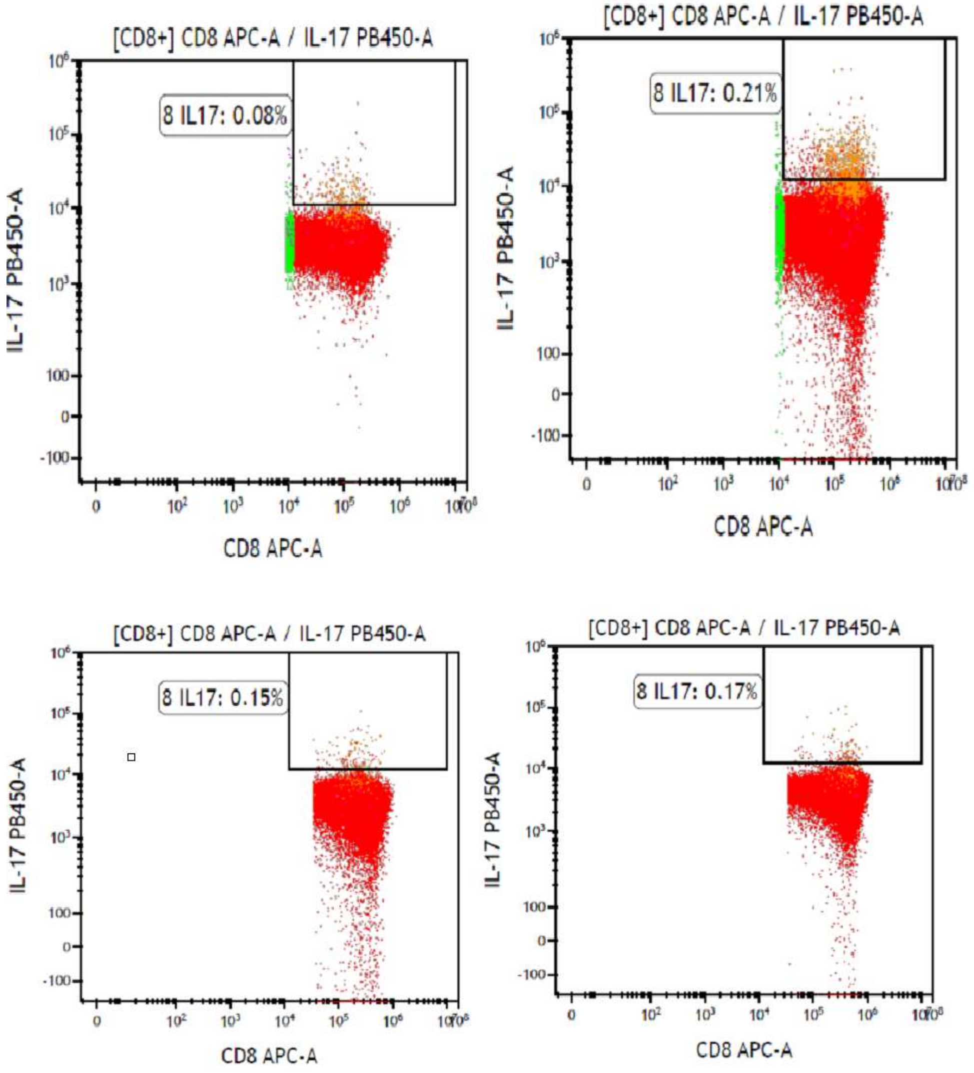
[CD8+] CD8 APC-A / IL-17 PB450-A
8 IL17: 0.08%
IL-17 PB450-A
CD8 APC-A
[CD8+] CD8 APC-A / IL-17 PB450-A
8 IL17: 0.21%
IL-17 PB450-A
CD8 APC-A
[CD8+] CD8 APC-A / IL-17 PB450-A
8 IL17: 0.15%
IL-17 PB450-A
CD8 APC-A
[CD8+] CD8 APC-A / IL-17 PB450-A
8 IL17: 0.17%
IL-17 PB450-A
CD8 APC-A

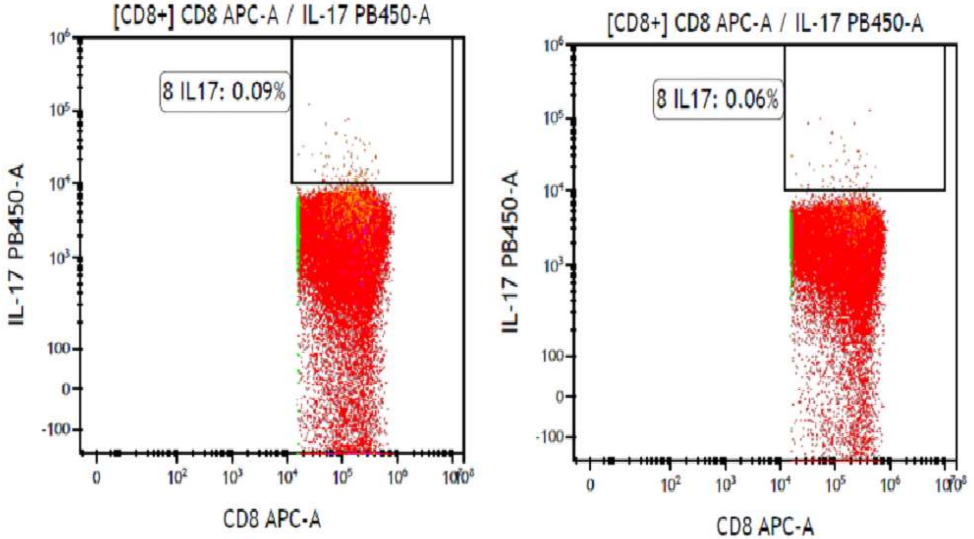
[CD8+] CD8 APC-A / IL-17 PB450-A
8 IL17: 0.09%
IL-17 PB450-A
CD8 APC-A
[CD8+] CD8 APC-A / IL-17 PB450-A
8 IL17: 0.06%
IL-17 PB450-A
CD8 APC-A

Panel D.

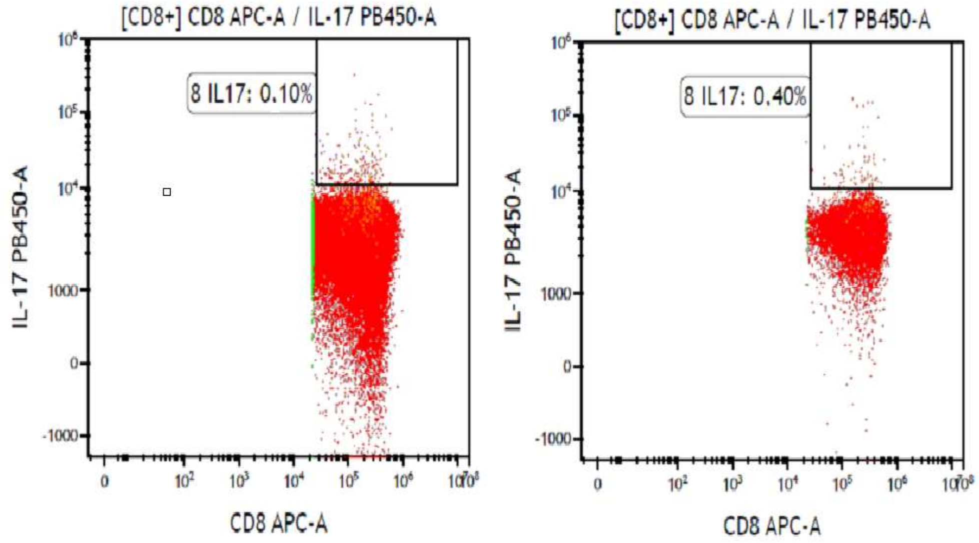
[CD8+] CD8 APC-A / IL-17 PB450-A
8 IL17: 0.10%
IL-17 PB450-A
CD8 APC-A
[CD8+] CD8 APC-A / IL-17 PB450-A
8 IL17: 0.40%
IL-17 PB450-A
CD8 APC-A

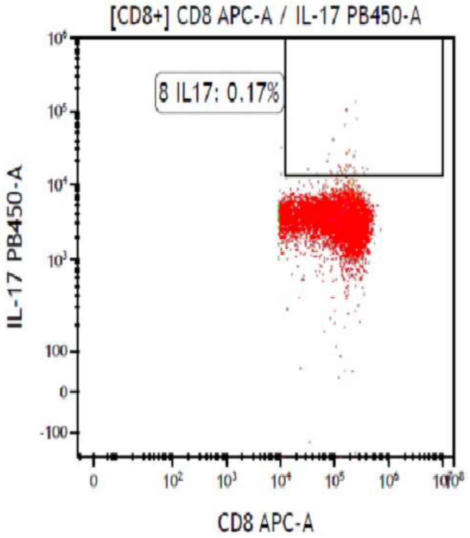
[CD8+] CD8 APC-A / IL-17 PB450-A
8 IL17: 0.17%
IL-17 PB450-A
CD8 APC-A

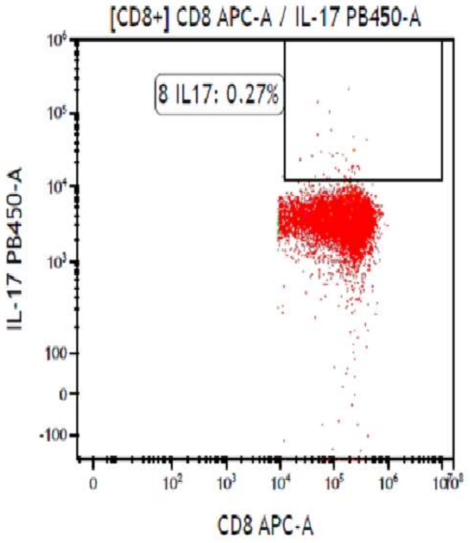
[CD8+] CD8 APC-A / IL-17 PB450-A
8 IL17: 0.27%
IL-17 PB450-A
CD8 APC-A

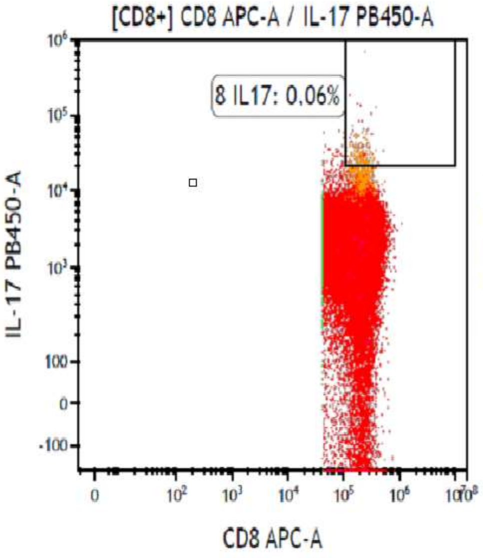
[CD8+] CD8 APC-A / IL-17 PB450-A
8 IL17: 0,06%
IL-17 PB450-A
CD8 APC-A

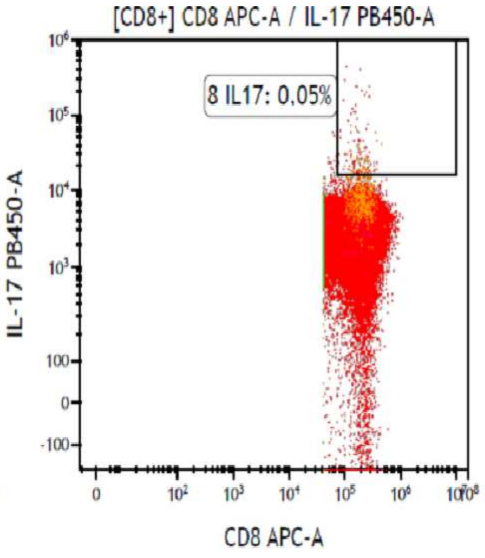
[CD8+] CD8 APC-A / IL-17 PB450-A
8 IL17: 0,05%
IL-17 PB450-A
CD8 APC-A

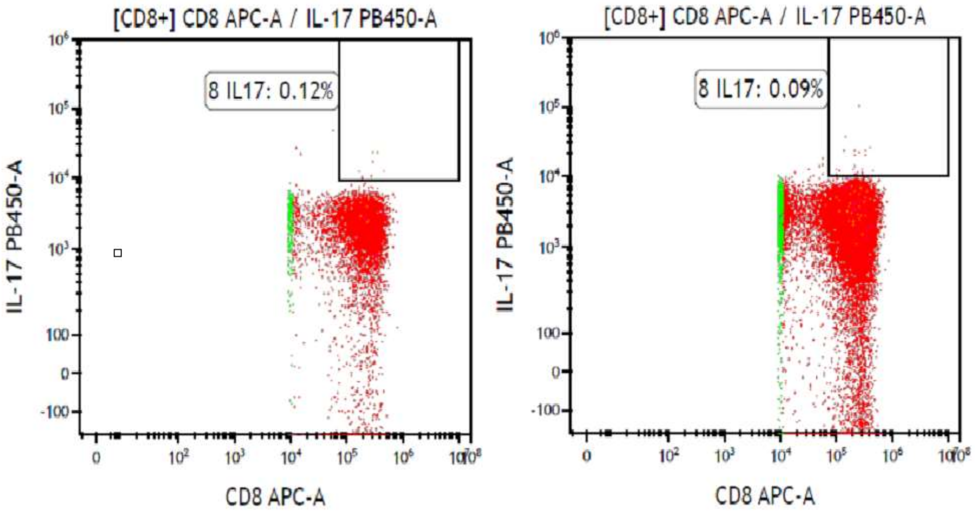

Supplementary Figure S3. Flow cytometry gating strategy for SARS-CoV-2 Spike-protein specific PBMCs expression of CD8^{+}IFNγ in stimulated (left) and unstimulated (right) samples of each MISC_A (Panel A), MISC_C (Panel B) and COVID-19 (Panel C).

Panel A.

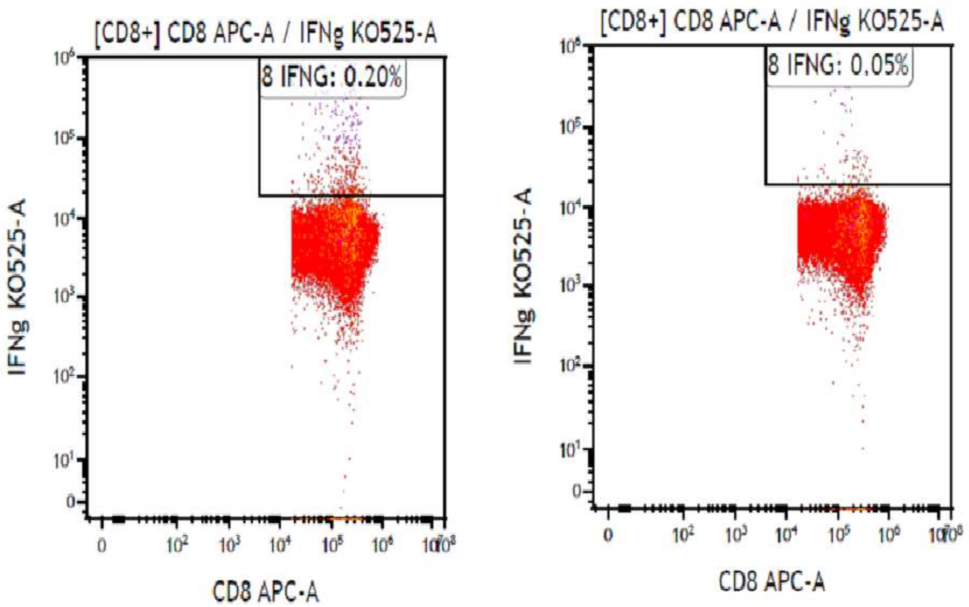
[CD8+] CD8 APC-A / IFNg KO525-A
8 IFNG: 0.20%
IFNg KO525-A
CD8 APC-A
[CD8+] CD8 APC-A / IFNg KO525-A
8 IFNG: 0.05%
IFNg KO525-A
CD8 APC-A

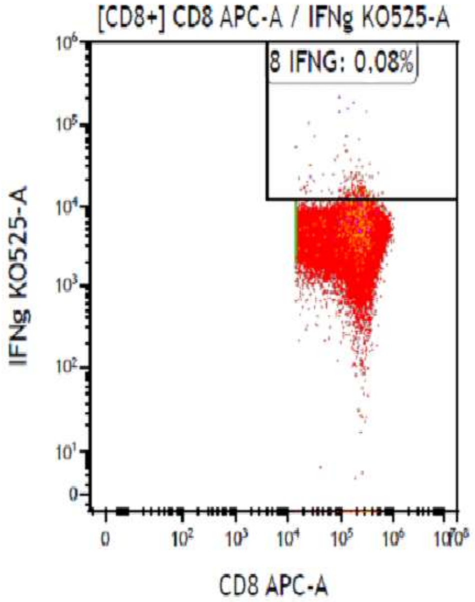
[CD8+] CD8 APC-A / IFNg KO525-A
8 IFNG: 0,08%
IFNg KO525-A
CD8 APC-A

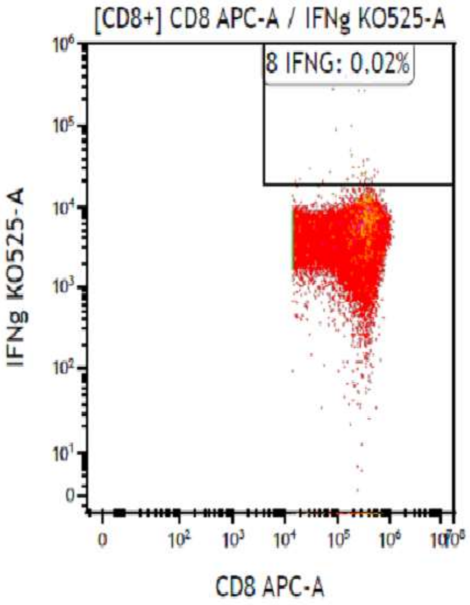
[CD8+] CD8 APC-A / IFNg KO525-A
8 IFNG: 0,02%
IFNg KO525-A
CD8 APC-A

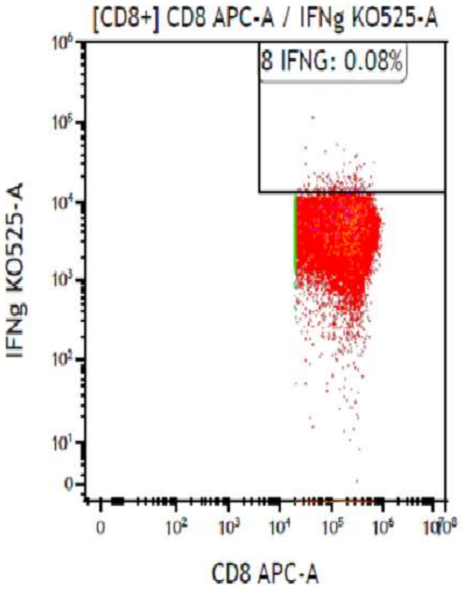
[CD8+] CD8 APC-A / IFNg KO525-A
8 IFNG: 0.08%
IFNg KO525-A
CD8 APC-A

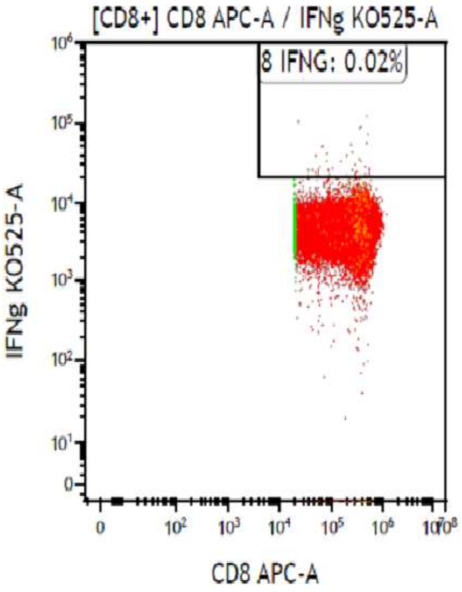
[CD8+] CD8 APC-A / IFNg KO525-A
8 IFNG: 0.02%
IFNg KO525-A
CD8 APC-A

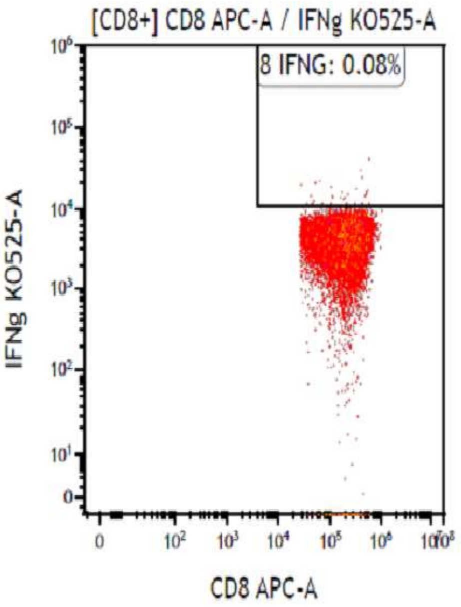
[CD8+] CD8 APC-A / IFNg KO525-A
8 IFNG: 0.08%
IFNg KO525-A
CD8 APC-A

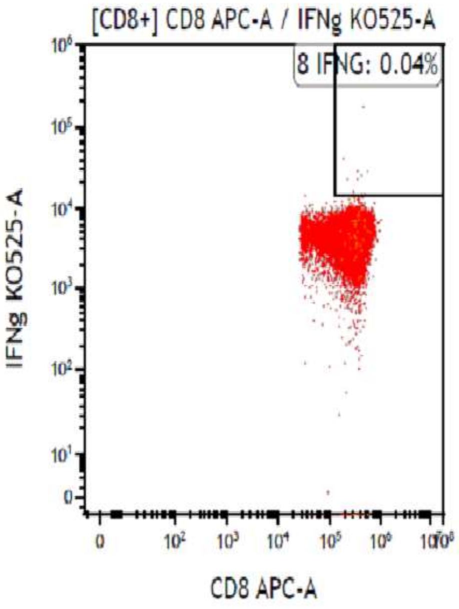
[CD8+] CD8 APC-A / IFNg KO525-A
8 IFNG: 0.04%
IFNg KO525-A
CD8 APC-A

Panel B.

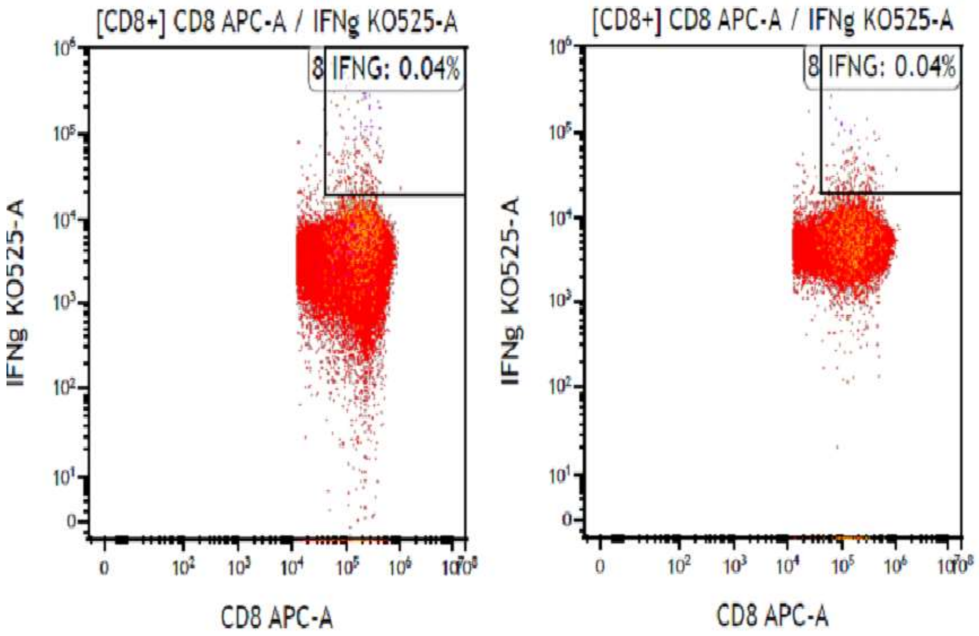
[CD8+] CD8 APC-A / IFNg KO525-A
8 IFNG: 0.04%
IFNg KO525-A
CD8 APC-A
[CD8+] CD8 APC-A / IFNg KO525-A
8 IFNG: 0.04%
IFNg KO525-A
CD8 APC-A

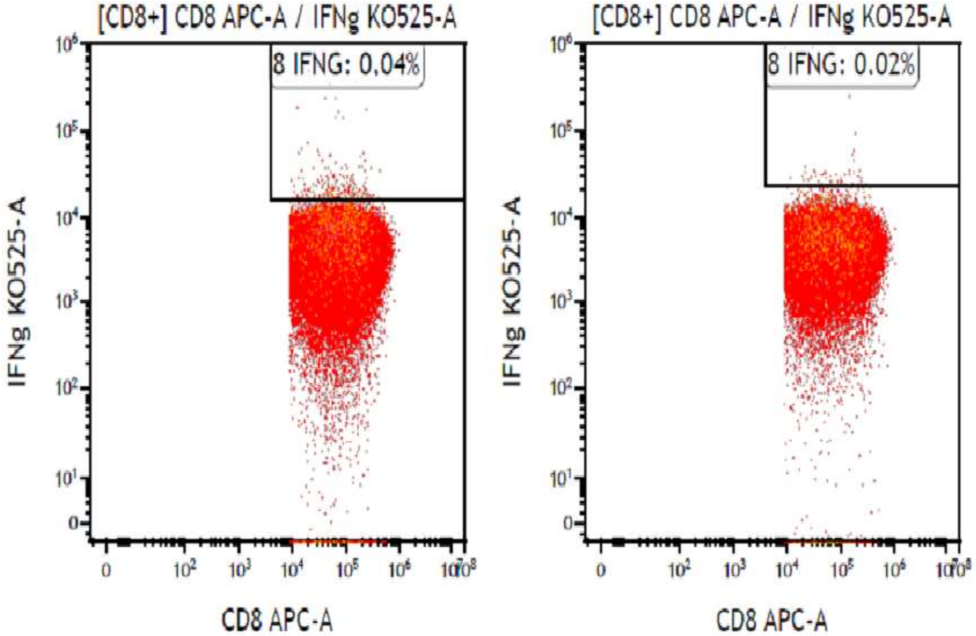

[CD8+] CD8 APC-A / IFNg KO525-A
8 IFNG: 0,04%
IFNg KO525-A
CD8 APC-A
[CD8+] CD8 APC-A / IFNg KO525-A
8 IFNG: 0,02%
IFNg KO525-A
CD8 APC-A

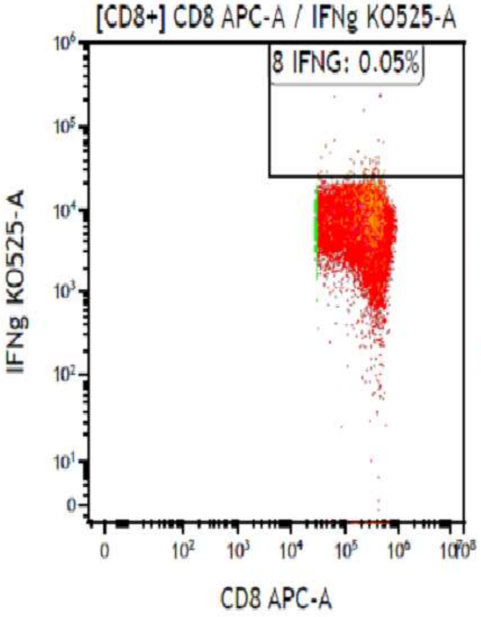
[CD8+] CD8 APC-A / IFNg KO525-A
8 IFNG: 0.05%
IFNg KO525-A
CD8 APC-A

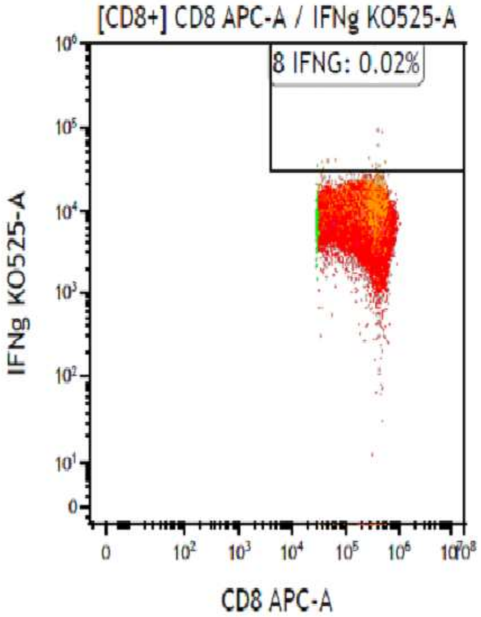
[CD8+] CD8 APC-A / IFNg KO525-A
8 IFNG: 0.02%
IFNg KO525-A
CD8 APC-A

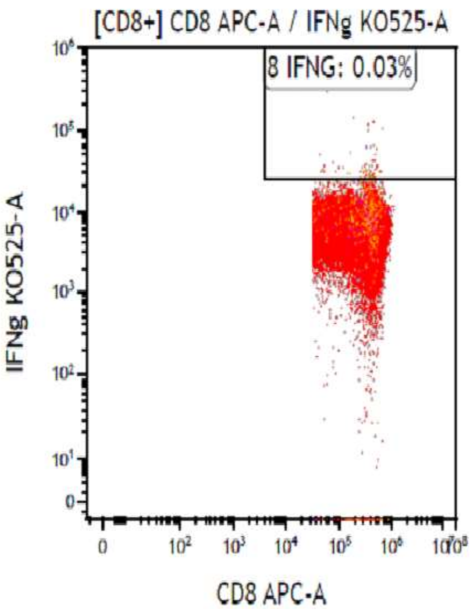
[CD8+] CD8 APC-A / IFNg KO525-A
8 IFNG: 0.03%
IFNg KO525-A
CD8 APC-A

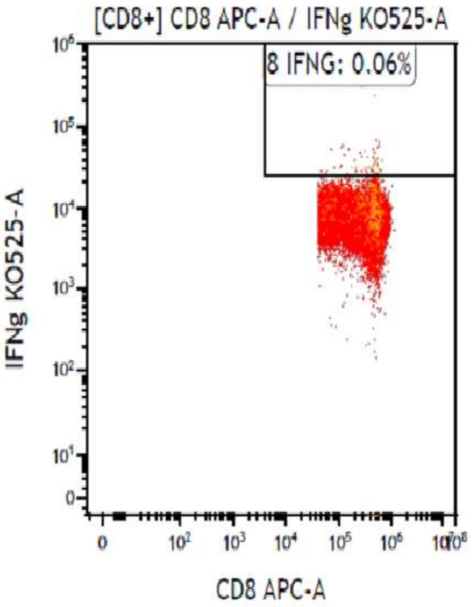
[CD8+] CD8 APC-A / IFNg KO525-A
8 IFNG: 0.06%
IFNg KO525-A
CD8 APC-A

Panel C.

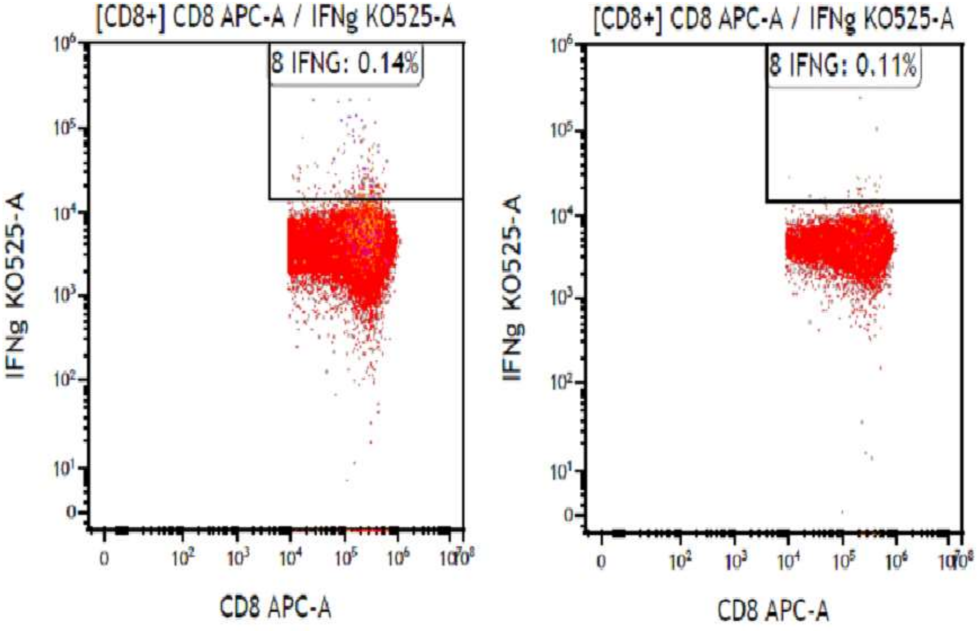
[CD8+] CD8 APC-A / IFNg KO525-A
8 IFNG: 0.14%
IFNg KO525-A
CD8 APC-A
[CD8+] CD8 APC-A / IFNg KO525-A
8 IFNG: 0.11%
IFNg KO525-A
CD8 APC-A

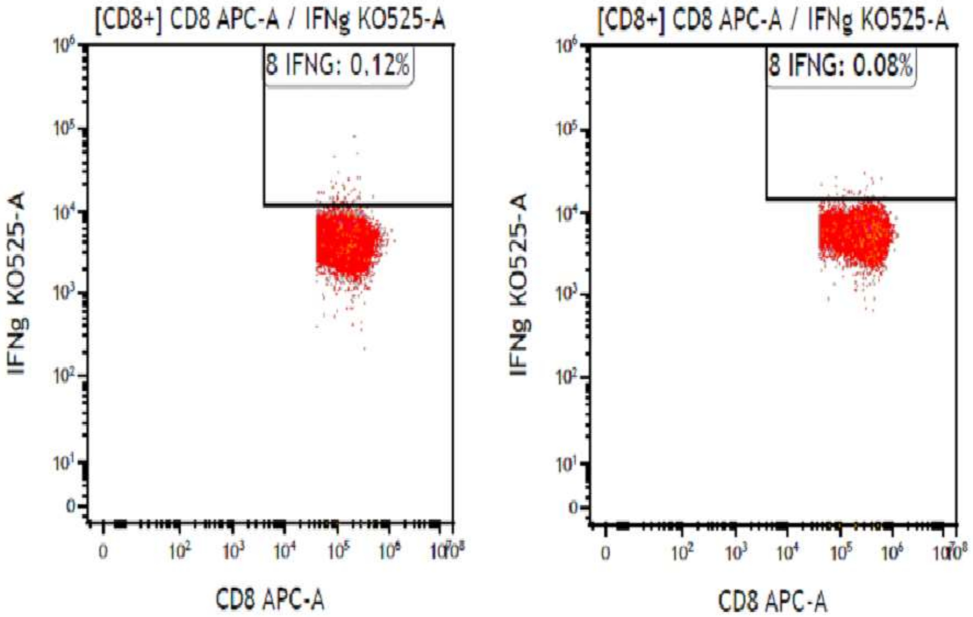
[CD8+] CD8 APC-A / IFNg KO525-A
8 IFNG: 0.12%
IFNg KO525-A
CD8 APC-A
[CD8+] CD8 APC-A / IFNg KO525-A
8 IFNG: 0.08%
IFNg KO525-A
CD8 APC-A

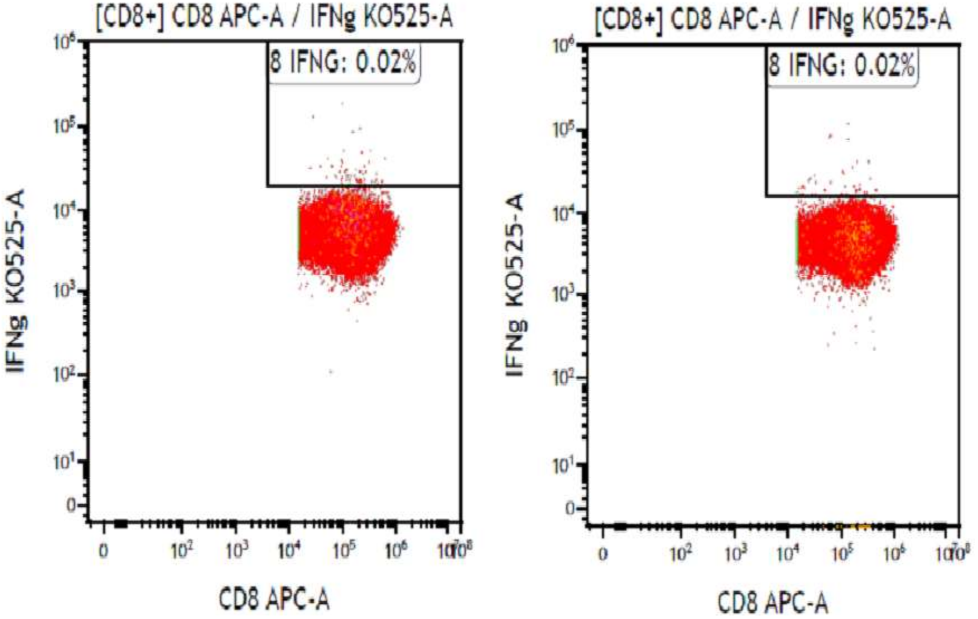
[CD8+] CD8 APC-A / IFNg KO525-A
8 IFNG: 0.02%
IFNg KO525-A
CD8 APC-A
[CD8+] CD8 APC-A / IFNg KO525-A
8 IFNG: 0.02%
IFNg KO525-A
CD8 APC-A

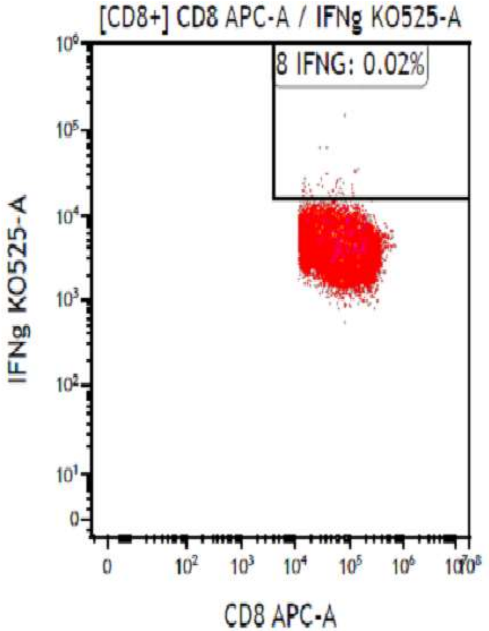
[CD8+] CD8 APC-A / IFNg KO525-A
8 IFNG: 0.02%
IFNg KO525-A
CD8 APC-A
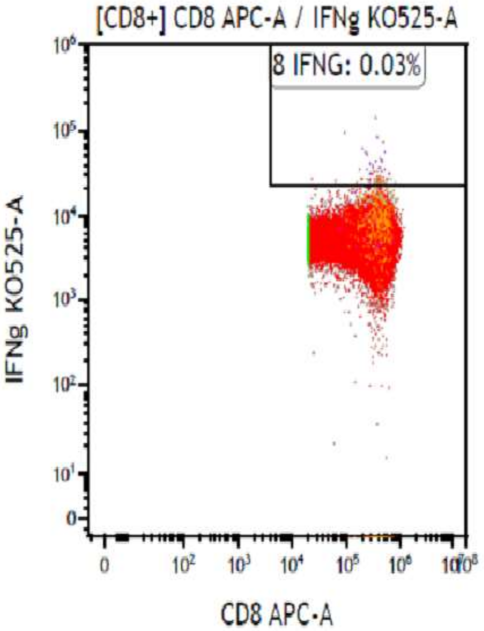
[CD8+] CD8 APC-A / IFNg KO525-A
8 IFNG: 0.03%
IFNg KO525-A
CD8 APC-A

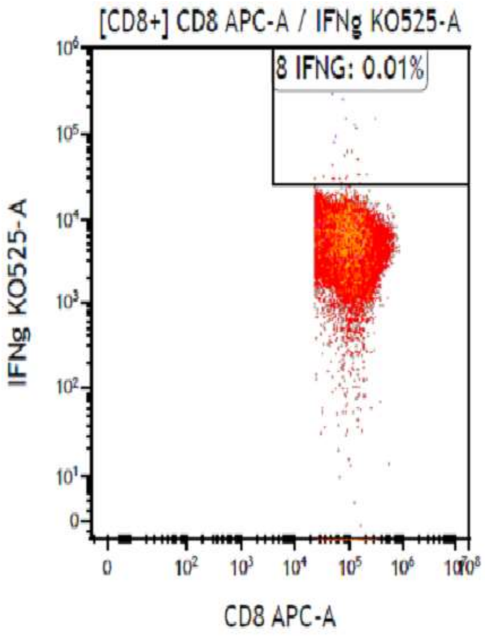
[CD8+] CD8 APC-A / IFNg KO525-A
8 IFNG: 0.01%
IFNg KO525-A
CD8 APC-A

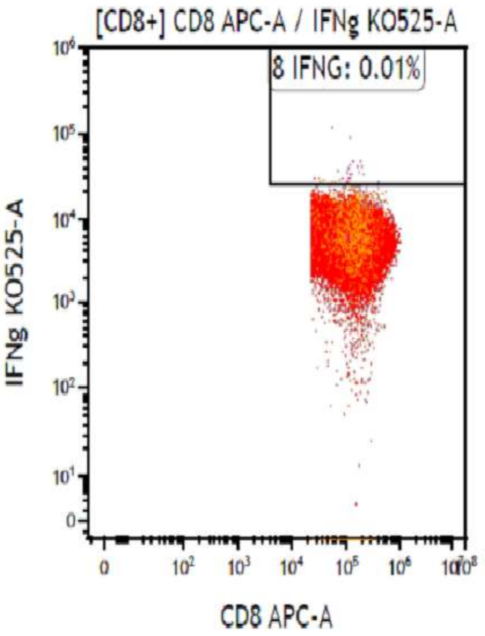
[CD8+] CD8 APC-A / IFNg KO525-A
8 IFNG: 0.01%
IFNg KO525-A
CD8 APC-A

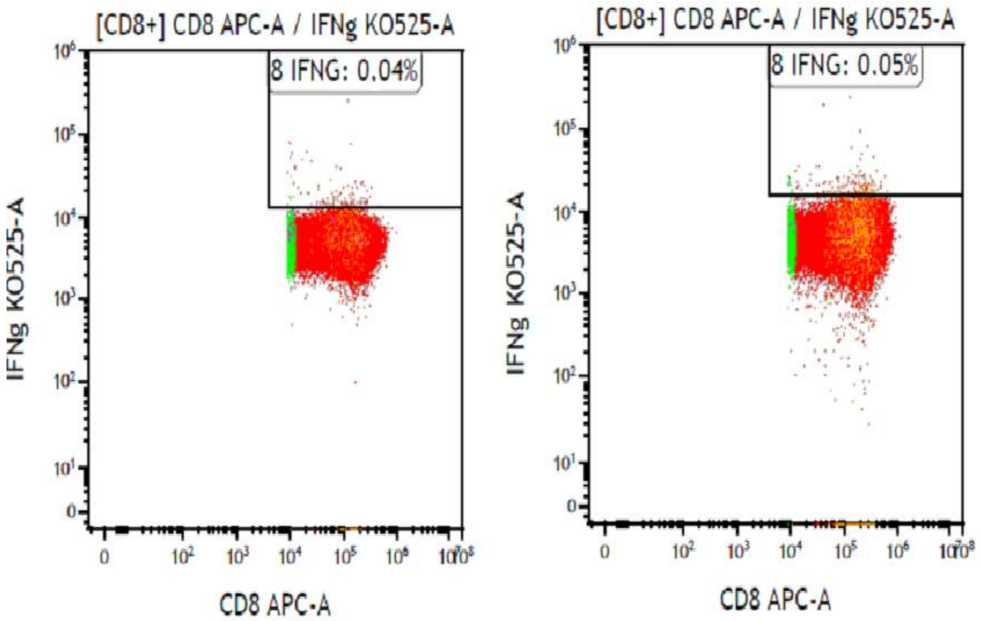
[CD8+] CD8 APC-A / IFNg KO525-A
8 IFNG: 0.04%
IFNg KO525-A
CD8 APC-A
[CD8+] CD8 APC-A / IFNg KO525-A
8 IFNG: 0.05%
IFNg KO525-A
CD8 APC-A

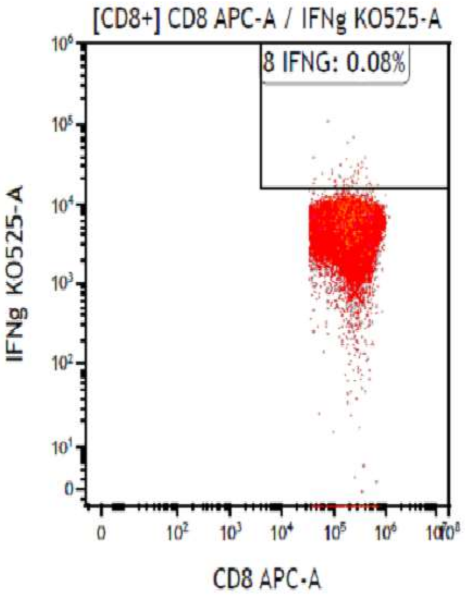
[CD8+] CD8 APC-A / IFNg KO525-A
8 IFNG: 0.08%
IFNg KO525-A
CD8 APC-A

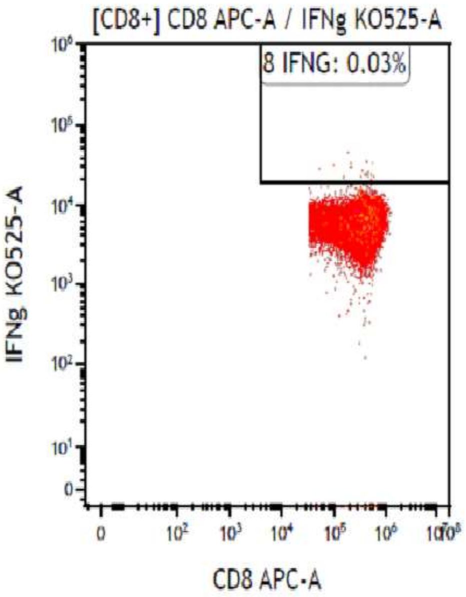
[CD8+] CD8 APC-A / IFNg KO525-A
8 IFNG: 0.03%
IFNg KO525-A
CD8 APC-A

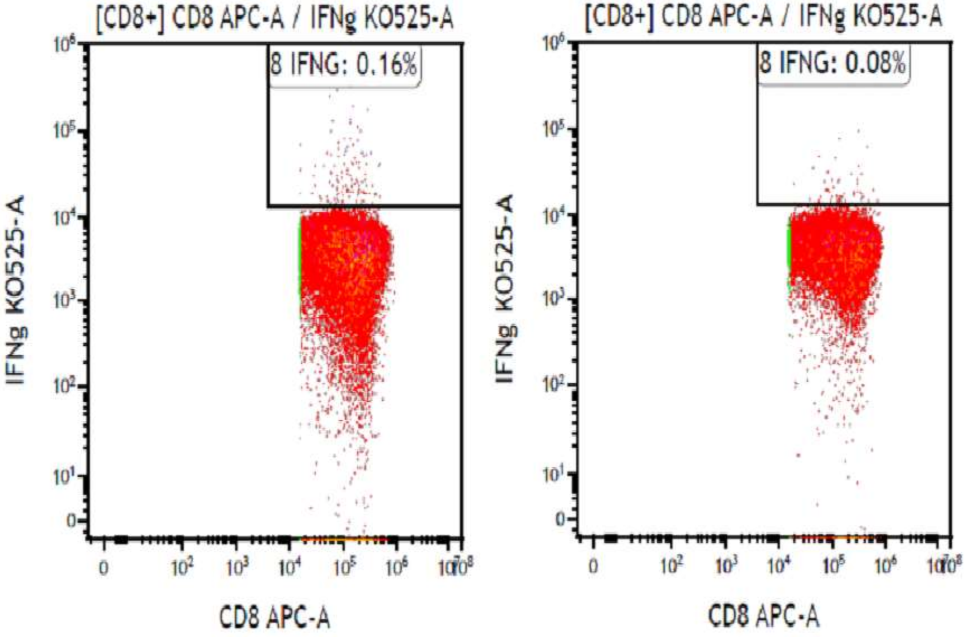
[CD8+] CD8 APC-A / IFNg KO525-A
8 IFNG: 0.16%
IFNg KO525-A
CD8 APC-A
[CD8+] CD8 APC-A / IFNg KO525-A
8 IFNG: 0.08%
IFNg KO525-A
CD8 APC-A

Panel D.

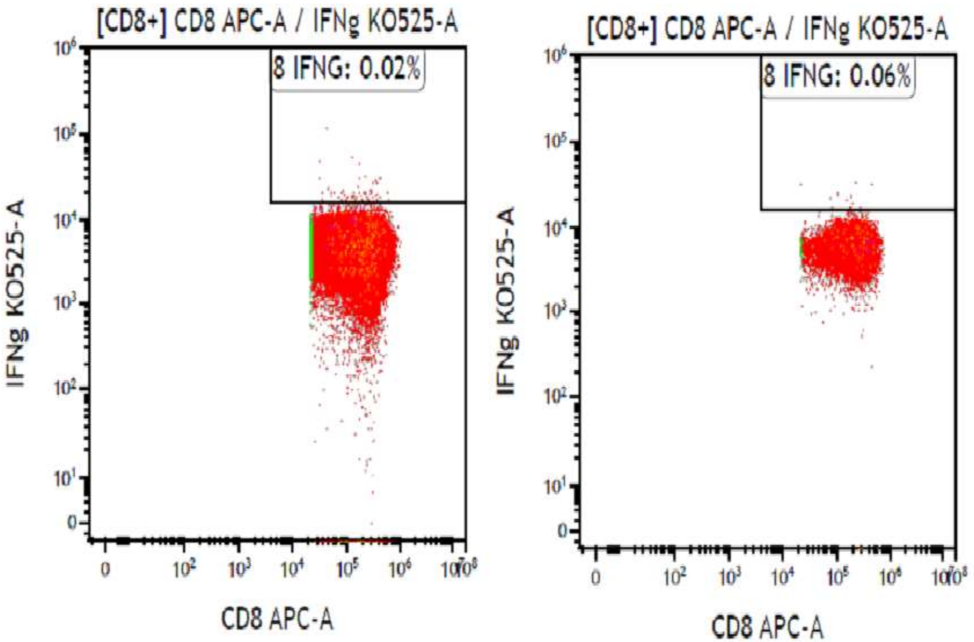
[CD8+] CD8 APC-A / IFNg KO525-A
8 IFNG: 0.02%
IFNg KO525-A
CD8 APC-A
[CD8+] CD8 APC-A / IFNg KO525-A
8 IFNG: 0.06%
IFNg KO525-A
CD8 APC-A

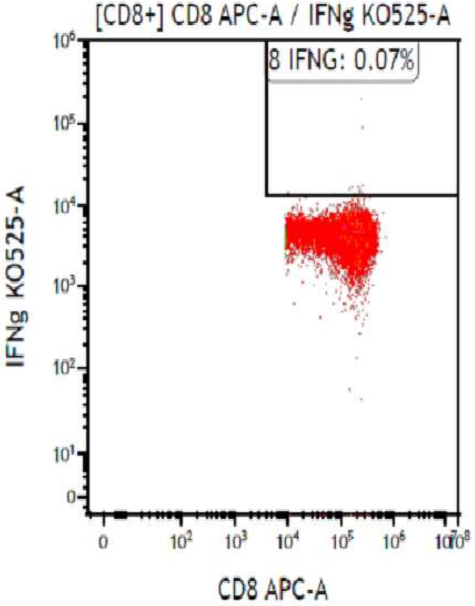
[CD8+] CD8 APC-A / IFNg KO525-A
8 IFNG: 0.07%
IFNg KO525-A
CD8 APC-A

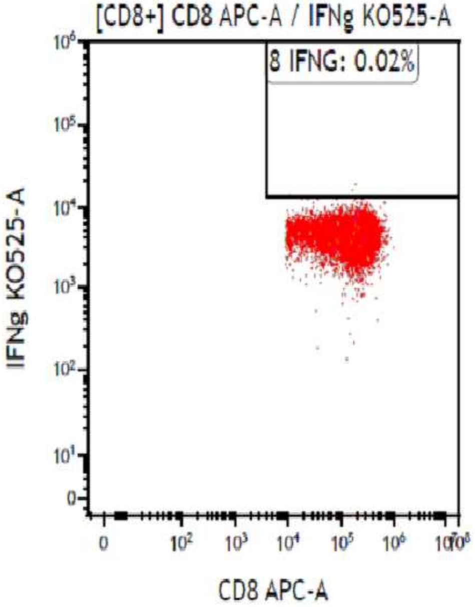
[CD8+] CD8 APC-A / IFNg KO525-A
8 IFNG: 0.02%
IFNg KO525-A
CD8 APC-A

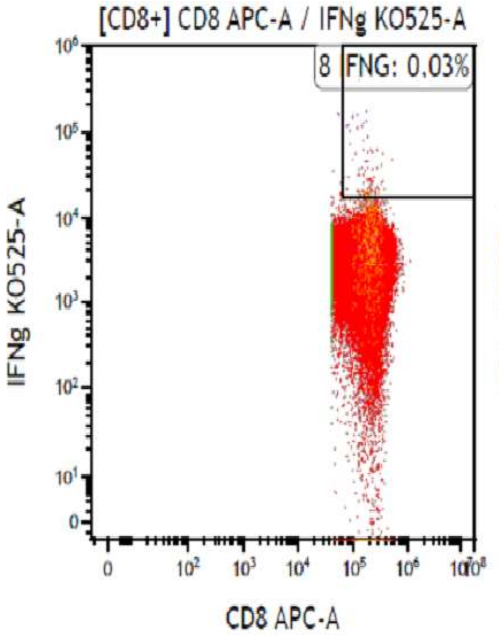
[CD8+] CD8 APC-A / IFNg KO525-A
8 IFNG: 0.03%
IFNg KO525-A
CD8 APC-A

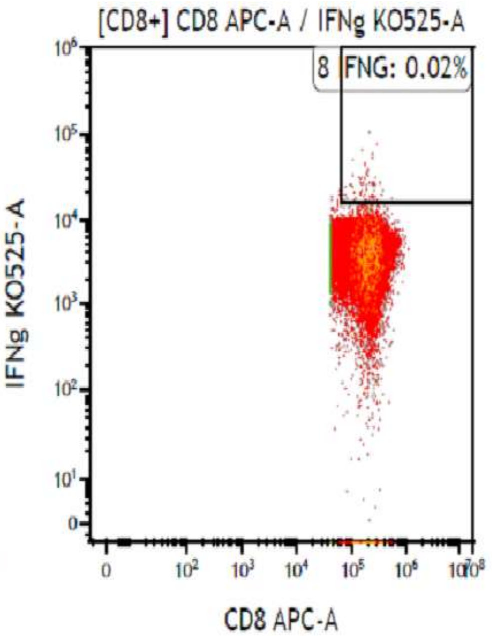
[CD8+] CD8 APC-A / IFNg KO525-A
8 IFNG: 0.02%
IFNg KO525-A
CD8 APC-A

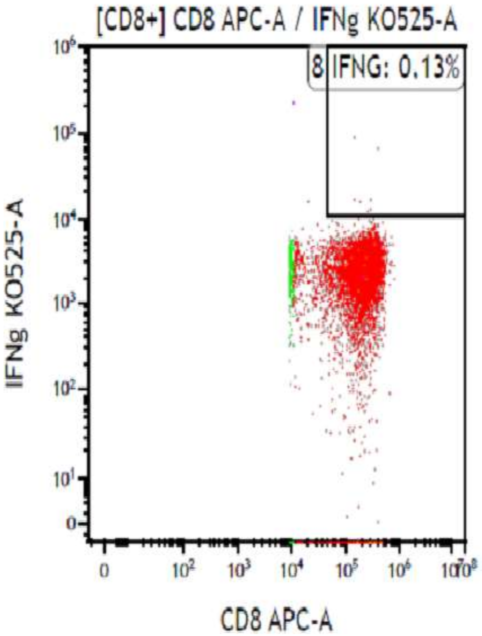
[CD8+] CD8 APC-A / IFNg KO525-A
8 IFNG: 0.13%
IFNg KO525-A
CD8 APC-A

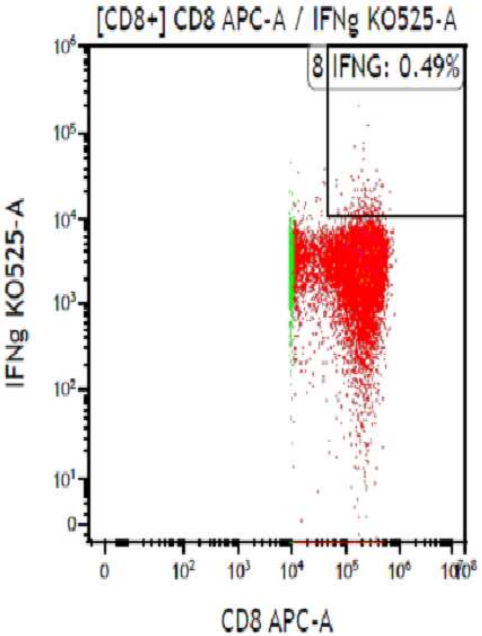
[CD8+] CD8 APC-A / IFNg KO525-A
8 IFNG: 0.49%
IFNg KO525-A
CD8 APC-A
